# Synthesis of Pore‐Wall‐Modified Stable COF/TiO_2_ Heterostructures via Site‐Specific Nucleation for an Enhanced Photoreduction of Carbon Dioxide

**DOI:** 10.1002/advs.202300073

**Published:** 2023-03-25

**Authors:** Akkammagari Putta Rangappa, Dharani Praveen Kumar, Khai H. Do, Jinming Wang, Yuexing Zhang, Tae Kyu Kim

**Affiliations:** ^1^ Department of Chemistry Yonsei University Seoul 03722 Republic of Korea; ^2^ College of Chemistry and Chemical Engineering Dezhou University Dezhou 253023 China

**Keywords:** CO_2_ reduction, covalent organic framework, N—Ti—O bond, photocatalysis, pore‐wall modification, site‐specific nucleation

## Abstract

Constructing stable heterostructures with appropriate active site architectures in covalent organic frameworks (COFs) can improve the active site accessibility and facilitate charge transfer, thereby increasing the catalytic efficiency. Herein, a pore‐wall modification strategy is proposed to achieve regularly arranged TiO_2_ nanodots (≈1.82 nm) in the pores of COFs via site‐specific nucleation. The site‐specific nucleation strategy stabilizes the TiO_2_ nanodots as well as enables the controlled growth of TiO_2_ throughout the COFs’ matrix. In a typical process, the pore wall is modified and site‐specific nucleation is induced between the metal precursors and the organic walls of the COFs through a careful ligand selection, and the strongly bonded metal precursors drive the confined growth of ultrasmall TiO_2_ nanodots during the subsequent hydrolysis. This will result in remarkably improved surface reactions, owing to the superior catalytic activity of TiO_2_ nanodots functionalized to COFs through strong N—Ti—O bonds. Furthermore, density functional theory studies reveal that pore‐wall modification is beneficial for inducing strong interactions between the COF and TiO_2_ and results in a large energy transfer via the N—Ti—O bonds. This work highlights the feasibility of developing stable COF and metal oxide based heterostructures via organic wall modifications to produce carbon fuels by artificial photosynthesis.

## Graphical abstract

The site‐specific nucleation strategy enables controlled growth of TiO_2_ throughout the covalent organic frameworks’ (COFs) matrix. This results in remarkably improved surface reactions, owing to the superior catalytic activity of TiO_2_ nanodots functionalized to COFs through N—Ti—O bonds. This work highlights the significance of an appropriate active site architecture in improving the photochemical behaviors and CO_2_ reduction reaction (CO_2_RR) performance.

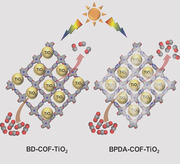

## 1 Introduction

Global attention has been drawn to the energy crisis and environmental issues caused by excessive CO_2_ emissions.^[^ ^1^, ^2^, ^3^ ^]^ In response to natural carbon limitations, the collective research focus has shifted to the development of effective methods for converting CO_2_ into value‐added products powered by inexhaustible solar light.^[^ ^1^, ^4^, ^5^, ^6^ ^]^ Various molecular‐ or semiconductor‐based systems have been established for CO_2_ photoreduction, but the presently known photosystems still face certain challenges such as low solar‐to‐chemical conversion efficiencies, poor selectivity, insufficient stability, unavoidable H_2_‐evolving reactions, and an unclear mechanism.^[^ ^1^, ^4^, ^7^, ^8^, ^9^, ^10^, ^11^ ^]^ These problems are mainly attributed to the poor CO_2_ adsorption, rapid charge recombination, and inappropriate architecture of active sites.^[^ ^8^, ^12^, ^13^ ^]^ To achieve this elusive goal, it is essential to introduce a rationally designed photocatalytic system that selectively and efficiently converts CO_2_ under solar light.

Over the last two decades, various advanced porous materials encompassing covalent organic frameworks (COFs),^[^ ^9^, ^13^, ^14^ ^]^ metal–organic frameworks,^[^ ^15^, ^16^, ^17^, ^18^, ^19^ ^]^ porous organic polymers,^[^ ^1^, ^20^ ^]^ zeolites,^[^ ^21^ ^]^ and other such materials^[^ ^15^, ^22^, ^23^, ^24^, ^25^, ^26^, ^27^, ^28^ ^]^ have been designed and explored for transforming CO_2_ molecules by organic reactions, hydrogenation, and photo‐ and electrocatalytic reduction reactions. The well‐defined structures of these crystalline porous species are advantageous for correlating the structure with CO_2_ conversion property from the atomic as well as molecular perspective. Among them, COFs have garnered significant interest because of their ability to broaden the precision of molecular chemistry to extended frameworks. Moreover, COFs are also considered as promising candidates for artificial photocatalysis.^[^ ^5^, ^9^, ^13^, ^14^, ^29^, ^30^ ^]^ Most COFs possess large surface areas and rich nitrogen atoms in their skeletons, which are beneficial for CO_2_ adsorption—one of the primary steps in CO_2_ photoreduction. These COFs offer *π*‐conjugated skeletons and long‐range ordered channels that facilitate an excellent light absorption, charge separation, and charge transportation.^[^ ^5^, ^9^, ^13^, ^14^, ^29^ ^]^ Therefore, COFs could be the ideal metal‐free organic semiconductors for artificial photosynthesis. Several studies have demonstrated the benefit of COFs in photocatalysis;^[^ ^5^, ^9^, ^13^, ^14^, ^29^ ^]^ however, the great potential of COFs as photocatalysts for CO_2_ reduction is now less appreciated, as more COF materials need to be discovered.

It is well known that the separation efficiency of photoinduced charge carriers in semiconductor photocatalysis plays a key role in determining the photocatalyst's performance. To improve the performance, porous materials are used as supports that encapsulate the additional active sites, which effectively serve as synergistic catalysts for CO_2_ reduction reaction (CO_2_RR).^[^ ^4^, ^9^, ^31^, ^32^ ^]^ Recently, significant efforts have been dedicated to improve the photostability and activity of COFs by decorating metal nanoparticles as additional active sites in COF matrices. These decorated metal nanoparticles can accelerate the charge separation as well as provide abundant active sites. For example, Zhang's group integrated Pt or Pd nanoparticles into thioether‐containing COFs for improving their catalytic performance.^[^ ^33^ ^]^ Guo et al. loaded Ru nanoparticles on ketoamine‐based COFs,^[^ ^9^ ^]^ resulting in a significantly enhanced photocatalytic CO_2_RR under visible light. Furthermore, some descriptors unraveled a strategy for encapsulating ultrasmall bimetallic clusters (Pd–In) to enhance the CO_2_RR performance and yield alcohols and others product.^[^ ^1^, ^8^, ^29^, ^34^, ^35^, ^36^, ^37^ ^]^ Despite these advances, the influence of these metal nanoparticles confined in COF matrices on the photoreduction performance still lacks a profound understanding.

In the majority of previously reported studies, a simple growth of metal nanoparticles in COF matrices was employed, which may unexpectedly interfere with the photocatalytic reactions. Because there is no control over the particles formed in COFs, simple pore‐filling methods can damage the inherent porous structures, thereby breaking the electron delocalization channels between the metal active sites and COF's extended conjugation; this results in a significant loss of electrical conductivity. Additionally, several Schottky junctions are expected between the uncontrolled nanoparticles and the COFs. These junctions can severely compromise the electrical pathways (*π*–d orbital overlap) and thus result in a limited efficiency.^[^ ^8^ ^]^ Moreover, during catalytic reactions, the weakly bonded particles tend to agglomerate, which limits their long‐term practical photocatalytic applications. Despite the demonstration of mono‐ and bimetallic nanoparticle‐decorated COFs, the lack of a reasonable mechanism for the formation of nanoparticles in COF pores remains an unresolved issue.^[^ ^9^, ^31^ ^]^ As a result, it is both desirable and difficult to construct noble‐metal‐free nanoparticles on COFs with a precise composition control, high uniformity, an excellent stability, and a narrow size distribution. To overcome these challenges and successfully functionalize nanodots in COFs without significant conductivity losses, it is necessary to develop a carefully designed CO_2_ photoreduction system and analyze it in detail.

Construction of coordinated photosensitizers (COFs) and active sites (metal nanoparticles) combines the advantages of delocalization of photoexcited charge carriers, light absorption, and photocatalytic performances.^[^ ^33^ ^]^ Hence, spatial confinement of additional active sites in COF matrices, to prevent their conductivity loss because of uncontrolled and agglomerated nanoparticles, is thus desired for achieving a high CO_2_ photoreduction efficiency. Bearing this in mind, our ultimate goal is to construct a stable COF/TiO_2_ heterojunction by the controlled growth of metal nanodots (TiO_2_) within well‐designed COFs for CO_2_ photoreduction. Thus, it is assumed that the photogenerated electrons originating from the COF can be injected into the TiO_2_ active sites through the electron delocalization channels to initiate CO_2_ reduction under solar light. To the best of our knowledge, this is the first report on a metal oxide nanodot‐filled COF‐based photocatalyst fabricated by a site‐specific nucleation process for CO_2_ photoreduction.

In this study, a highly stable pore‐wall‐modified COF heterostructure was synthesized by inserting TiO_2_ nanodots into the COF pores, according to the strategy of ligand‐induced site‐specific nucleation. The strong coordinated bonds linked the metal precursors and bipyridyl units together, and the subsequent hydrolysis process produced ultrasmall (≈1.82 nm) and regularly arranged TiO_2_ nanodots in the pores of the COF matrix. The obtained COF/TiO_2_ with strong N—Ti—O bonds exhibited an excellent CO_2_RR performance, which is twice that of the simple pore‐filled COF. This remarkable performance improvement can be attributed to the efficient charge transfer among the N—Ti—O bonds. These results provide significant perspective on charge‐transfer efficiencies, which are crucial for designing stable COF‐based photocatalysts by replacing the noble metal active sites with ultrasmall metal oxide nanodots.

## 2 Results and Discussion

**Scheme 1:**
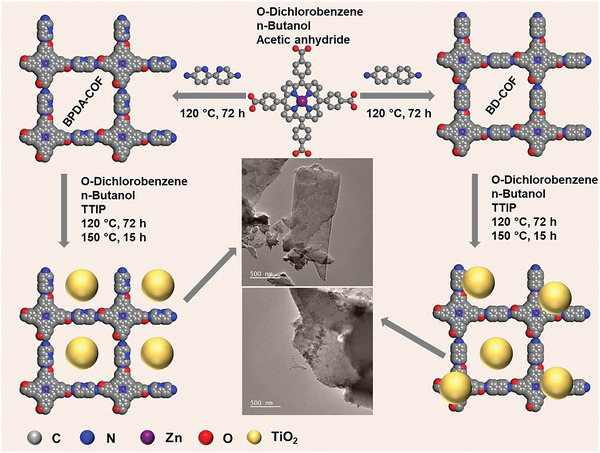
Schematic showing the synthesis of the COF heterostructures and their corresponding TEM images.

COF/TiO_2_ heterostructures were synthesized by in situ hydrolyzing titanium isopropoxide (TTIP) onto the COF matrix (**Scheme** **1**). To investigate the role of an appropriate active site architecture, two different ligands (benzidine (BD) and (2,2″‐bipyridine)‐5,5″‐diamine (BPDA)) were used to induce site‐specific nucleation as well as to stabilize the TiO_2_ nanodots. The powder X‐ray diffraction (PXRD) patterns shown in Figure S1 (Supporting Information) reveal that both the COF and COF/TiO_2_ heterostructures exhibit identical diffraction peaks with reduced peak intensities. This result indicates that the crystallinity is slightly reduced when the TiO_2_ nanodots are incorporated. The anatase phase of TiO_2_ was represented in the synthesized TiO_2_ by the crystal planes at 25.3°, 38°, 48°, 54°, and 62.5°.^[^ ^35^ ^]^ The observation of similar intensive lattice planes for BD—COF—TiO_2_ confirms the presence of TiO_2_ nanoparticles on the COF matrix. Though the peaks corresponding to TiO_2_ are not clearly observed in the patterns of BPDA—COF—TiO_2_, the highlighted and zoomed region for BPDA—COF—TiO_2_ confirms the presence of broadened characteristic peaks of TiO_2_. This result suggests the presence of controlled and stabilized ultrasmall TiO_2_ nanodots, which were formed by the N—Ti—O bonds initiated by the site‐specific nucleation. This hypothesis is further supported by the X‐ray photoelectron spectroscopy (XPS), energy‐dispersive X‐ray spectroscopy (EDX), and Fourier transform infrared (FTIR) analysis, which are discussed later in this paper.

The successful introduction of Zn into porphyrin (Ppy) ring and the formation of COF/TiO_2_ heterostructures were confirmed by nuclear magnetic resonance (NMR) spectroscopy, ultraviolet–visible diffuse reflectance spectroscopy (DRS), FTIR spectroscopy, and XPS. The NMR spectra of free Ppy show a peak at −2.9 ppm, which can be attributed to pyrrolic hydrogen; the disappearance of the peak upon the formation of the metalloporphyrin Zn—Ppy confirms the successful synthesis of Zn—Ppy (Figure S2, Supporting Information).^[^ ^38^ ^]^ Moreover, the FTIR analysis confirmed the presence of a strong peak corresponding to N—Zn at ≈998 cm^−1^, suggesting the successful preparation of Zn—Ppy (Figure S3, Supporting Information).^[^ ^39^ ^]^ Intensive peaks corresponding to the C=O (≈1600 cm^−1^), N—H (≈1410 cm^−1^), and C—N (≈1335 cm^−1^) bonds are observed in COF spectra, confirming the formation of an organic framework with C=O stretch, C—N stretch, and N—H bending vibrations, respectively (Figure S4a, Supporting Information).^[^ ^40^, ^41^, ^42^, ^43^ ^]^ In addition, the peak corresponding to —OH stretch in Zn—Ppy is slightly shifted to the lower‐frequency region and justifies the successful formation of amide bonds with —NH stretching vibrations (Figure S4b, Supporting Information). All the peaks that originate from a typical organic framework are observed in the spectra of the COF/TiO_2_ heterostructures, signifying the stability of the COF structure under TiO_2_ loading.^[^ ^40^, ^41^, ^42^, ^43^ ^]^ Moreover, the site‐specific nucleated heterostructure (BPDA—COF—TiO_2_) reveals an additional peak at 900 cm^−1^ associated with the N—Ti—O bonds (Figure S4, Supporting Information).^[^ ^39^ ^]^ The N—Ti—O bonds formed as a result of the COFs’ pore‐wall modification with a BPDA ligand containing bipyridyl units and the Ti metal precursors at the framework. These bipyridyl units are more likely to form strong coordination bonds with Ti metal precursors, resulting in the nucleation of metal nanoparticles and the formation of N—Ti—O bonds, which facilitated a stabilized and controlled TiO_2_ growth throughout the COFs’ matrix.

**Figure 1:**
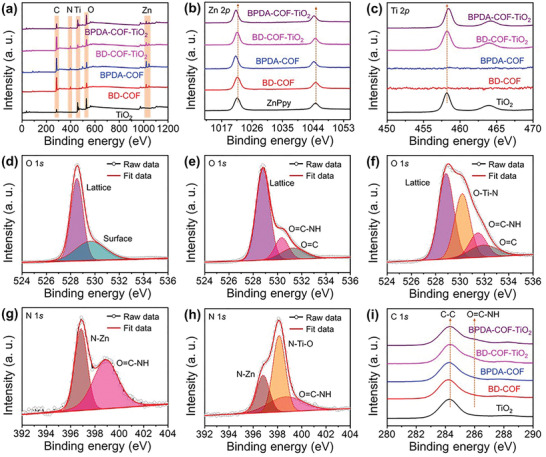
a) XPS survey spectra and b,c) high‐resolution XPS spectra of b) Zn 2p and c) Ti 2p. Fitted data of the high‐resolution O 1s spectra for d) TiO_2_, e) BD—COF—TiO_2_, and f) BPDA—COF—TiO_2_. Fitted data of the high‐resolution N 1s spectra for g) BD—COF—TiO_2_, and h) BPDA—COF—TiO_2_. i) High‐resolution XPS profile of C 1s.

The electronic structures of the COF samples and their composites were investigated by XPS. The XPS survey spectra (**Figure** **1**a) confirmed the existence of Ti, O, Zn, N, and C in the COF/TiO_2_ composite, while Zn, N, and C were found in the COF samples and Ti and O in the synthesized TiO_2_. The high‐resolution Zn 2p spectra (Figure 1b) of BPDA—COF and BPDA—COF—TiO_2_ show a decreased binding energy (BE) of ≈0.4 eV with Zn—Ppy indicating the existence of strong pull‐and‐push electron interactions between the Zn sites and the bipyridyl units of the ligand. However, the BE of Ti 2p (Figure 1c) in the case of BPDA—COF—TiO_2_ increases by ≈0.4 eV compared with that of pure TiO_2_. This increase in the Ti 2p BE can be attributed to the existence of strong N—Ti—O bonds accompanied by site‐specific nucleation between the metal precursor and the bipyridyl units of the ligand. Notably, the BE values of BD—COF and BD—COF—TiO_2_ show a negligible change, unlike those of the synthesized Zn—Ppy and TiO_2_, implying a poor interaction between the COF and TiO_2_ when simple pore‐filling methods are implemented. The opposite BE shifts in the case of Zn 2p and Ti 2p demonstrate that a strong metal‐to‐metal charge transfer occurs via the N—Ti—O bonds as an interfacial charge‐transfer bridge.^[^ ^44^, ^45^ ^]^ The high‐resolution O 1s spectrum of TiO_2_ exhibits two typical BE peaks (Figure 1d) at 528.5 and 529.6 eV, which can be ascribed to the interstitial and adsorbed oxygen of TiO_2_.^[^ ^46^ ^]^ Moreover, the O 1s spectra obtained from BD—COF—TiO_2_ (Figure 1e) could be deconvoluted into three BE peaks at 528.5, 530.3, and 531.3 eV, which correspond to the interstitial oxygen of TiO_2_, formation of organic framework with O=C—NH, and O=C bonds, respectively.^[^ ^45^ ^]^ In contrast to TiO_2_ and BD—COF—TiO_2_, the O 1s spectrum of BPDA—COF—TiO_2_ exhibits a new peak at 530.2 eV (Figure 1f), which can be ascribed to the N—Ti—O bonds induced by the site‐specific nucleation.

In addition, the N 1s peak is evident in the XPS spectra of all the samples, except TiO_2_ (Figure 1a). Because of the two different environments of N atoms in the free Ppy ring, it displays two peaks (=N— and —NH—).^[^ ^39^ ^]^ When Zn is evenly coordinated with the N atoms, a single peak is observed, which validates the formation of Zn—Ppy (Figure S5, Supporting Information).^[^ ^39^ ^]^ Moreover, the high‐resolution N 1s spectrum of BD—COF—TiO_2_ (Figure 1g) can be deconvoluted into two peaks at 396.8 and 399 eV, which can be assigned to N—Zn and O=C—NH in the composite.^[^ ^44^ ^]^ Notably, the N 1s spectra of BPDA—COF—TiO_2_ (Figure 1h) show an extra peak at 398.1 eV, which can be ascribed to the N—Ti—O bond, while those at 396.8 and 399 eV correspond to N—Zn and O=C—NH, respectively. Finally, the high‐resolution C 1s spectrum (Figure 1i) shows a peak at 286 eV, which is attributed to O=C—NH in the COFs and their heterostructures.^[^ ^44^, ^45^ ^]^ These results validate the successful synthesis of a stable COF/TiO_2_ heterostructure through the N—Ti—O bonds induced by the site‐specific nucleation process in BDPA—COF—TiO_2_.

**Figure 2:**
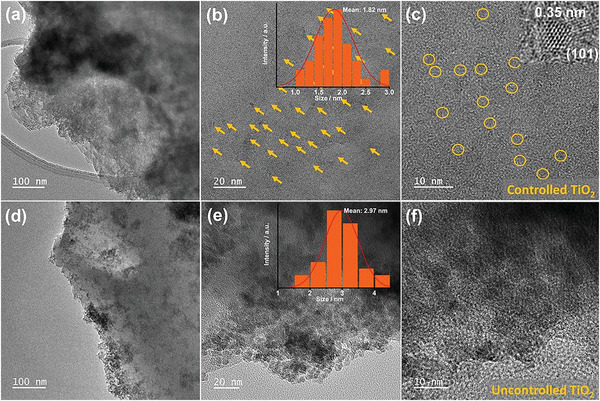
TEM images and (inset) particle size distribution of TiO_2_: a–c) BPDA—COF—TiO_2_ and d–f) BD—COF—TiO_2_ composites.

To further explore the microstructure of the catalysts, we analyzed the morphology of the TiO_2_, COF, and COF/TiO_2_ heterostructures by using a field‐emission scanning electron microscope (FESEM) and transmission electron microscope (TEM). The synthesized pure TiO_2_ exhibits sphere‐like structures (Figure S6, Supporting Information), whereas the COFs show stacked sheet‐like structures (Figures S7 and S8, Supporting Information). The TEM images (**Figure** **2**) show that the pore‐wall modification induced a site‐specific nucleation, which facilitated the confined growth and regular arrangement of TiO_2_ nanoparticles into the pores of the COF matrix (Figure 2a–c; Figure S9a–d, Supporting Information). Figure 2c (inset) confirms the presence of TiO_2_ nanodots confined in the COFs’ matrix by displaying a lattice distance of 0.35 nm belonging to the TiO_2_ (101) plane. The average size of these ultrasmall TiO_2_ dots is ≈1.82 nm (Figure 2b). The simple pore‐filled BD—COF—TiO_2_ demonstrates that the majority of the TiO_2_ is deposited on the surface or at the edges with an average diameter of ≈2.97 nm (Figure 2d–f; Figure S10a–d, Supporting Information). This result highlights the importance of ligand selection for obtaining appropriate and regularly arranged stable architectures. This hypothesis is further supported by the EDX analysis results, which reveal that BPDA—COF—TiO_2_ had a homogeneous distribution of Ti and O; this confirms the controlled growth of ultrasmall TiO_2_ nanodots throughout the COFs’ matrix (Figure S9e, Supporting Information). However, the dispersion of Ti and O in BD—COF—TiO_2_ is uneven, and most of the TiO_2_ is present at the edges as agglomerated particles (Figure S10e, Supporting Information). These results indicate that stable heterostructures were successfully fabricated via the site‐specific nucleation.

**Figure 3:**
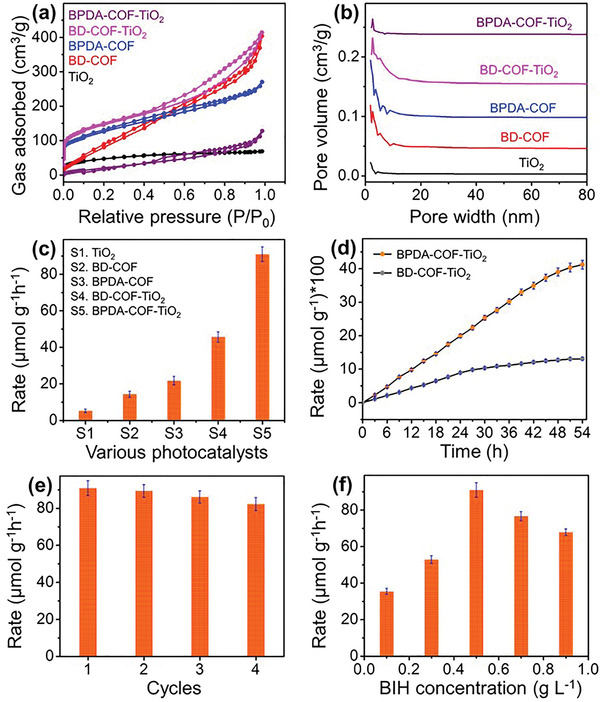
a) Liquid N_2_ adsorption–desorption isotherms and b) pore size distribution of TiO_2_, COFs, and their composites. c) Average rate of CO generation on various photocatalysts for 5 h. d) Stability of the CO production rates on the COF/TiO_2_ heterostructures under continuous light irradiation. e) Long‐time stability study of the BPDA—COF—TiO_2_ heterostructure with light on/off cycle mode process. f) Effect of BIH concentration on the photoreduction of CO_2_ using BPDA—COF—TiO_2_.

The marked changes in the micromorphology of the samples caused some observable distinctions in the liquid N_2_ adsorption–desorption experiment results, ascribable to the appropriate architecture of the COF/TiO_2_ heterostructures (**Figure** **3**a). The COF samples exhibited type IV‐like isotherms with an insignificant H1 hysteresis loop at high partial pressures (*P*/*P*_0_ > 0.6) and an H2‐like hysteresis loop at low partial pressures (*P*/*P*_0_ = 0.5). These loops can be attributed to the capillary condensation of the N_2_ molecules among the well‐defined cylindrical pore channels accompanied by stacking in the COFs.^[^ ^47^, ^48^ ^]^ Because of the presence of a nonrigid particle like TiO_2_ in the COF matrix, the simple pore‐filled heterostructure BD—COF—TiO_2_ exhibits an H3 hysteresis loop. The presence of narrow slit‐like pores generated by the controlled growth of TiO_2_ caused the site‐specific nucleated heterostructure BPDA—COF—TiO_2_ to show an H4 hysteresis loop, thereby confirming the existence of a microporous region (<2 nm).^[^ ^47^, ^48^ ^]^ As shown in Figure 3a, the specific surface areas for BD—COF and BPDA—COF are 288 and 348 m^2^ g^−1^, respectively. Since most of the TiO_2_ nanoparticles are deposited on the edges of the COF in BD—COF—TiO_2_, the specific surface area increases from 288 to 416 m^2^ g^−1^. Interestingly, BPDA—COF—TiO_2_ shows a reduced specific surface area (71 m^2^ g^−1^), indicating that TiO_2_ nanodots were successfully grown in the pores of the COF matrix.^[^ ^8^, ^34^ ^]^ This point is further proven by the Barrett–Joyner–Halenda (BJH) pore size distribution (Figure 3b), which shows that the size distribution exhibited by BPDA—COF—TiO_2_ is narrower than that of BPDA—COF. However, BD—COF—TiO_2_ shows a negligible change in the pore size distribution when compared with that of BD—COF. This result proves the successful growth of ultrasmall TiO_2_ dots via site‐specific nucleation in BPDA—COF, consistent with the results of the XRD and TEM analyses.

The reaction medium was first optimized using various additives to achieve selective photoreduction of CO_2_ (Figure S11, Supporting Information). The control experiments showed that only CO could be produced from the synthesized nanocomposites in the mixture of acetonitrile and 1,3‐dimethyl‐2‐phenyl‐2,3‐dihydro‐1*H*‐benzo[d]imidazole (BIH) during photoreaction of CO_2_ irradiated by a 150 W Xe lamp (Figure S11, Supporting Information). The comparative experiments indicated that no reduced products of CO_2_ could be detected from the photoreaction system without a photocatalyst, light, and CO_2_, demonstrating that the obtained CO originated from the photoreaction under the solar light (Figure S12, Supporting Information). To optimize the CO_2_ photoreduction conditions over the COF/TiO_2_ heterostructures, the effects of appropriate active site architecture were initially surveyed. As shown in Figure 3c, pure TiO_2_ produces only small amounts of CO (5 µmol g^−1^ h^−1^). Moreover, the pre‐eminent visible‐light absorption of the COFs significantly enhances the CO generation rate to 14 and 22 µmol g^−1^ h^−1^ for BD—COF and BPDA—COF, respectively. Surprisingly, the COF/TiO_2_ composites outperform TiO_2_ and the free COFs in terms of activity, with evolving rates of 46 and 91 µmol g^−1^ h^−1^ for BD—COF—TiO_2_ and BPDA—COF—TiO_2_, respectively. Notably, the CO_2_RR performance of the regularly arranged TiO_2_ nanoparticles in the COF with site‐specific nucleation is two times higher than that of the simple pore‐filled heterostructure (BD—COF—TiO_2_). The CO evolution activity was improved ≈2.39 fold, from 38 to 91 µmol g^−1^ h^−1^ as the amount of TiO_2_ was raised from 5 to 20%, followed by a slightly declining trend with further increasing the TiO_2_ dosage to 30% (Figure S13, Supporting Information). The enhancement in the activity parallel to the increase in TiO_2_ filling could be ascribed to the TiO_2_ dots promoting the charge separation through N—Ti—O bonds for CO_2_ reduction. Conversely, excessive TiO_2_ loading will result in denser distribution of nanoparticles in the COFs’ matrix, which will then absorb and scatter the incident photons, weakening the TiO_2_ function. As shown in Figure 3d, long‐time photostability tests were also carried out for the BD—COF—TiO_2_ and BPDA—COF—TiO_2_ heterostructures. It can be seen that the prepared BPDA—COF—TiO_2_ photocatalyst exhibits a sustained photoactivity for ≈50 h, but the performance of the BD—COF—TiO_2_ catalyst slowly decays after ≈24 h. We believe that the presence of the strong N—Ti—O bonds in BPDA—COF—TiO_2_ resulted in its long‐term stability, and thus, BPDA—COF—TiO_2_ is suitable for long‐term CO_2_ reduction under solar light. In addition, the changes in the crystal structure and morphology of the composites were proven by XRD (Figure S14, Supporting Information) and TEM (Figures S15 and S16, Supporting Information) analyses. There are no obvious changes for fresh and retrieved samples, indicating that the composites are stable for long runs under solar light. It is evident that the presence of delocalization channels (N—Ti—O) in BPDA—COF—TiO_2_ promotes catalyst's durability over BD—COF—TiO_2_ for long‐term practical applications. Furthermore, the stability of the catalyst BPDA—COF—TiO_2_ was assessed in four consecutive cycles (Figure 3e), in which the reactor was vacuum‐treated and purged with CO_2_ after each reaction. An average CO production rate of 91.2% is maintained, indicating that the photocatalysts are stable over multiple cycles. The BIH concentration for the optimized CO_2_ reduction performance is 0.5 g L^−1^ (Figure 3f). These findings suggest that ligand selection to stabilize the TiO_2_ nanodots in a framework is critical for an efficient CO_2_RR, because the strong N—Ti—O bonds, which are formed through pore‐wall modification, act as bridges for the photogenerated charges and promote charge separation and transportation. As a result, majority of the electrons participate in the CO_2_RR. The photoactivity of CO_2_ reduction was compared with the previously reported COF‐ and TiO_2_‐based catalysts in Table S1 (Supporting Information).

**Figure 4:**
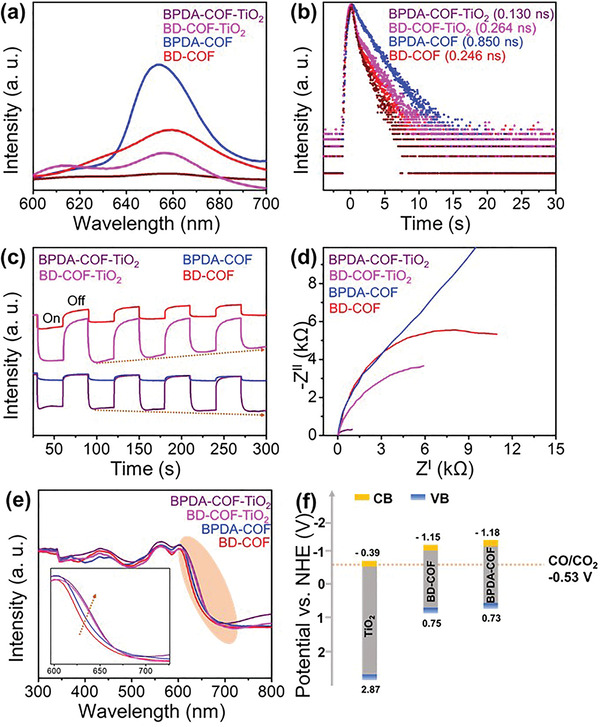
a) Photoluminescence spectra, b) TRFS, c) photocurrent response spectra, d) electrochemical impedance spectra, and e) DRS absorption profiles of BD—COF, BPDA—COF, BD—COF—TiO_2_, and BPDA—COF—TiO_2_, and f) schematic of the possible energy diagram of the TiO_2_ and COF nanocomposites.

Photocharge carrier behaviors were also investigated to gain deeper insights into the photoreaction mechanism underlying the enhanced CO_2_RR. The transfer pathway of the photoexcited charge carriers was revealed by photoluminescence (PL) spectroscopy. As shown in **Figure** **4**a, when excited by a 373 nm light, all the samples show intense PL emissions at 657 nm. The PL intensity of the COF/TiO_2_ heterostructures is lower than that of the bare COFs, indicating the existence of nonradiative pathways formed by the delocalization of the electrons from the COF onto the TiO_2_ active sites. Notably, the lowest PL intensity for BPDA—COF—TiO_2_ signifies the existence N—Ti—O bonds for a quick and an efficient electron transfer to TiO_2_.^[^ ^49^ ^]^ As evident from the femtosecond‐time‐resolved fluorescence spectra (TRFS), the PL lifetimes of BD—COF (0.246 ns) and BD—COF—TiO_2_ (0.264 ns) do not show any significant difference (Figure 4b). This indicates the absence of electron‐transfer channels between the two components. Clearly, the BPDA—COF—TiO_2_ composite exhibits a shorter lifetime (0.13 ns) than BPDA—COF (0.85 ns), confirming the rapid transfer of photogenerated charge carriers to the TiO_2_ active sites through the N—Ti—O bonds. Furthermore, charge separation efficiency was revealed by transient photocurrent response measurements (Figure 4c). The photocurrent for the COF/TiO_2_ heterostructures is higher than that of the simple COF samples. The transient photocurrent response measurements show that the BPDA—COF—TiO_2_ heterostructure exhibits a photocurrent similar to BD—COF—TiO_2_. However, BPDA—COF—TiO_2_ exhibits a stable photocurrent over time, indicating that the formation of the N—Ti—O bonds via the site‐specific nucleation process ensured a more efficient charge separation and transportation onto the TiO_2_ active sites. Moreover, electrochemical impedance spectroscopic measurements (Figure 4d) of the BPDA—COF—TiO_2_ system show the smallest semicircle, confirming a faster interfacial charge transfer onto the active sites via N—Ti—O bonds. Despite the fact that the BD—COF—TiO_2_ composite demonstrated a reasonable charge separation efficiency (Figure 4c), the interfacial charge transfer resistance is greater than that of BPDA—COF—TiO_2_. The absence of delocalization channels within the BD—COF—TiO_2_ matrix compromises the electrical pathways (*π*–d orbital overlap) that lead to higher interfacial charge‐transfer resistance (Figure 4d) and lower photocatalytic activity. These findings highlight the significance of active site architecture in improving the photochemical behaviors and CO_2_RR performance.

**Figure 5:**
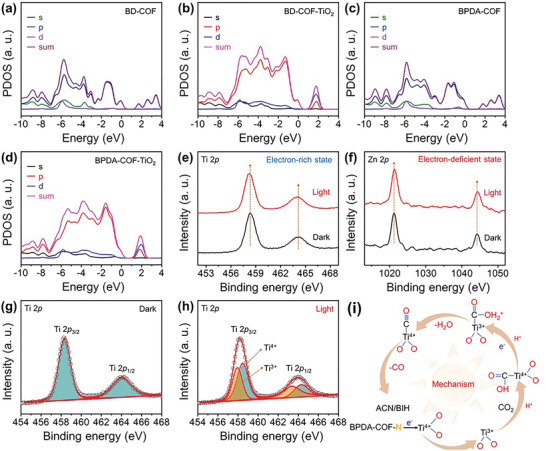
PDOS of the a) BD—COF, b) BD—COF—TiO_2_, c) BPDA—COF, and d) BPDA—COF—TiO_2_ nanocomposites. In situ XPS spectra of the high‐resolution e) Ti 2p, and f) Zn 2p for the BPDA—COF—TiO_2_ composites. Fitted data of the high‐resolution Ti 2p spectra for g) dark, and h) light. i) Photoreaction mechanism of CO_2_ reduction to CO on BPDA—COF—TiO_2_ composites.

Finally, to probe the photoreaction mechanism underlying the enhanced CO_2_RR activity of the COFs and their composites, optical absorption and energy band structures of the matrices were explored. The DRS spectra of Ppy exhibit the characteristic four peaks of Q bands, which turn to two peaks after Zn^2+^ introduction (Figure S17, Supporting Information).^[^ ^39^ ^]^ The presence of identical peaks in the DRS profiles of all the composites signifies the structural stability of Zn—Ppy during the preparation of the COF and COF/TiO_2_ heterostructures (Figure 4e). Because of the synergistic effect between the two components, the COF/TiO_2_ heterostructures exhibit an enhanced absorption in the visible region, compared with pure COF. It is obvious that among all the samples, BPDA—COF—TiO_2_ exhibits the best photoabsorption, which is responsible for its enhanced CO_2_RR performance under solar light. Tauc plots were derived from (*αhv*)*^n^* versus light energy (*hv*) (*n* = 2 for direct bandgap)^[^ ^15^ ^]^ to determine the bandgap energy (*E*_g_) values of the prepared composites (Figure S18, Supporting Information). We obtained *E*_g_ = 1.90 eV (BD—COF), 1.91 eV (BPDA—COF), 1.94 eV (BD—COF—TiO_2_), 1.86 eV (BPDA—COF—TiO_2_), and 3.24 eV (TiO_2_). Furthermore, the Mott–Schottky curves of the synthesized TiO_2_ nanodots display positive slopes (Figure S19, Supporting Information), suggesting their n‐type semiconducting behavior, with a flat band potential (*E*_fb_) of ≈−0.59 V versus Ag/AgCl (at 25 °C in saturated KCl solution, the electrode potential (*E^*θ*^*) = 0.198 V vs normal hydrogen electrode (NHE)).^[^ ^12^ ^]^ However, the COF structures exhibit negative slopes, implying p‐type characteristics, with *E*_fb_ values of ≈ 0.56 (BD—COF) and ≈0.54 (BPDA—COF) in the presence of Ag/AgCl. Following potential conversion from the reference Ag/AgCl to NHE,^[^ ^12^ ^]^ the *E*_fb_ values are estimated to be −0.39 V (TiO_2_), 0.75 V (BD—COF), and 0.73 V (BPDA—COF). Since the difference between the conduction band for n‐type semiconductors and the valence band for p‐type semiconductors is negligible, we obtain *E*_fb_ = *E*_CB_ and *E*_VB_ (where *E*_CB_ and *E*_VB_ denote the valance band and conduction band energies, respectively) for the n‐type and p‐type semiconductors, respectively. Further, based on the equation *E*_CB_ *= E*_VB_ − *E*_g_, the band positions were measured, and a relative potential energy diagram was drawn (Figure 4f) to demonstrate the feasibility of CO_2_ reduction to CO. To further reveal the effect of the ligand on the electronic band structure and contributions of each related orbital to the *E*_CB_ and *E*_VB_ values of these COF/TiO_2_‐based materials, their partial density of states (PDOS) were calculated by employing the first‐principles pseudopotential methods based on density functional theory (DFT). For the calculation, the Ti_16_O_32_ nanoparticles were placed in the pores of the COF to optimize the geometry (Figure S20, Supporting Information). As shown in **Figure** **5**, the PDOS corresponding to the COF (Figure 5a,c) shows that the top of the valance band is primarily derived from the 2p orbitals, whereas the bottom of the conduction band is mainly composed of relatively delocalized 3d and 2p orbitals. After the incorporation of TiO_2_ (Figure 5b,d), a noticeable change is observed in both the 3d and 2p orbitals because of the established interactions between the COF and TiO_2_ through the H—O (2.123 and 2.576 Å) and N—Ti (2.222 and 2.237 Å) bonds in BD—COF—TiO_2_ and BPDA—COF—TiO_2_, respectively (Figure S20, Supporting Information). The obtained Ti—N bond length is consistent with the previously reported results,^[^ ^50^, ^51^ ^]^ suggesting the formation of N—Ti—O bonds in BPDA—COF—TiO_2_ composite as confirmed by FTIR and XPS analyses. These interactions are beneficial for the injection of electrons onto the TiO_2_ active sites. Furthermore, the observed 3D charge density distribution in both the COF/TiO_2_ matrices confirms the efficient charge transfer of electrons onto the TiO_2_ active sites for CO_2_ reduction (Figure S20b,d, Supporting Information). Although there is no significant difference between the PDOS of the two COF/TiO_2_ composites, the BPDA—COF—TiO_2_ composite exhibits slightly higher density of states due to the existence of strong N—Ti—O bonds. In addition, the calculated binding energy between TiO_2_ and the BPDA ligand (−3.90 eV) is higher than that of the BD ligand (−2.38 eV) (Figure S20, Supporting Information), indicating stronger interactions between BPDA—COF and TiO_2_. These strong interactions are beneficial for the energy transfer via the N—Ti—O bonds.

**Scheme 2:**
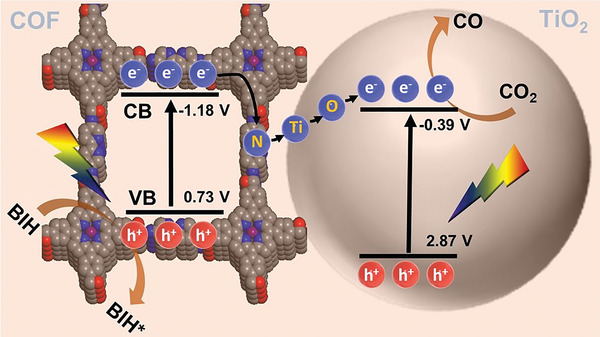
Schematic of the energy band structure and photoreaction mechanism on the COF/TiO_2_ heterostructure.

In situ techniques such as XPS and FTIR were used to investigate the structure functional relationships and the precise photocatalytic CO_2_ reduction mechanism. As illustrated in Figure 5e–h, when the BPDA—COF—TiO_2_ composite was exposed to the light illumination condition, the binding energy belong to Ti 2p (Figure 5e) and Zn 2p (Figure 5f) slightly shifted to the negative and positive sides, respectively. According to the electrostatic shielding effect, more outer electrons result in a lower binding energy, implying that the photogenerated electrons transfer from Zn—Ppy to TiO_2_ via N—Ti—O bonds to initiate the CO_2_ photoreduction.^[^ ^52^, ^53^, ^54^ ^]^ To make our above conjecture more explicit, the Ti 2p spectra of dark and light were accurately fitted (Figure 5g,h). The Ti 2p spectra obtained in the dark condition presented the peaks of Ti 2p_3/2_ (458.3 eV) and Ti 2p_1/2_ (464.0 eV) corresponding to Ti^4+^ states of TiO_2_ (Figure 5g). Notably, the Ti 2p spectra under the light could be deconvoluted into an extra peak (Figure 5h), belong to Ti^3+^ states of TiO_2_, suggesting the accumulation of photoexcited electrons on to TiO_2_ active sites.^[^ ^55^ ^]^ In addition, to track the reaction intermediates under the photocatalytic CO_2_ reduction, we carried out diffuse reflectance infrared Fourier transform spectroscopy (DRIFT). The results in Figure S21 (Supporting Information) presented the peaks at 1011, 1327, and 1362 cm^−1^ corresponding to H—C=O bending vibration of *CHO species, *COOH (OH) and *COOH (CO) vibrations, respectively. These species can be regarded as crucial intermediates during the photocatalytic CO_2_ reduction to CO or CH_4_.^[^ ^56^, ^57^, ^58^ ^]^ As the reaction proceeds, the intensity of *COOH intermediates over BPDA—COF—TiO_2_ surface was increased along with the existence of the new peak at (2127 cm^−1^) that is ascribable to *CO.^[^ ^56^, ^57^, ^58^ ^]^ No significant peaks were observed in the spectrum belong to other products,^[^ ^56^, ^57^, ^58^ ^]^ signifying that CO is the only product in the present system (Figure S11, Supporting Information). These findings suggest the promotion of photocatalytic CO_2_ reduction to CO on the surface of BPDA—COF—TiO_2_ under light (**Scheme** **2**).

Based on all experimental and theoretical evidence, including in situ analyses, a possible photoreaction mechanism can be elucidated as shown in Scheme 2 and Figure 5i. The strong pull‐and‐push interactions between the two components cause transfer of photogenerated electrons from the COF to the TiO_2_ catalytic sites via the N—Ti—O bonds, thus achieving a spatial separation. Upon light absorption by the BPDA—COF—TiO_2_ heterostructure, photogenerated electrons simultaneously appear on both the COF and TiO_2_ conduction bands. The oxidation of BIH molecule on Zn—Ppy units results in the reduction of Zn^II^ to Zn^I^, followed by the metal‐to‐metal charge transfer (Zn^I^ to Ti^4+^) with the assistance of the N—Ti—O bonds generated via the site‐specific nucleation process.^[^ ^59^ ^]^ The electrons accumulated on TiO_2_ can participate in the desired CO_2_RR to produce CO through an electron–proton (H^+^ from BIH) coupling pathway (Figure 5i). Moreover, the holes that are generated at the valence band of TiO_2_ are transferred to that of the COF and react with BIH present in the system, thereby realizing a successful CO_2_ photoreduction on the COF/TiO_2_ heterostructures.

## 3 Conclusion

In summary, a family of COF heterostructures based on a metalloporphyrin Zn—Ppy was prepared to unveil how the specific active site (TiO_2_) architecture influences the photoreduction properties of the COF. Experimental findings and DFT results showed that site‐specific nucleation results in structurally regulated active sites (TiO_2_) that are precisely confined within the COF matrix. The active‐site‐confined COF can be synthesized without causing any structural deformation in the COF. A stronger interaction between the COF and TiO_2_ was generated by the N—Ti—O bonds for an effective electron transfer, which enhanced the photocatalytic performance. By virtue of these advantages, the BPDA—COF—TiO_2_ heterostructure photocatalyzed CO_2_ reduction under solar light and yielded 91 µmol g^−1^ h^−1^ of CO, which was approximately two times higher than that obtained by using the simple pore‐filled heterostructure BD—COF—TiO_2_. Through this study, we experimentally and theoretically demonstrated the merits of pore‐wall modification in obtaining stable COF/TiO_2_ heterostructures, which serve as highly active catalytic sites as well as boost the charge separation efficiency via N—Ti—O bond formation. These findings on active site architecture in COF matrices are particularly useful for the development of non‐noble‐metal‐based COF heterostructures for sustained and stable CO_2_ reduction reactions under solar light.

## 4 Experimental Section

### Materials

Tetrakis (4‐carboxyphenyl) porphyrin, Zn acetate, BD, BPDA, TTIP, o‐dichlorobenzene, butanol, acetic anhydride, dichloromethane, methanol, dimethyl formamide (DMF), and deionized water were used. Free porphyrin was obtained from TCI chemicals, and all the other chemicals were purchased from Daejung chemicals and used without any further purification.

### Synthesis of Zn—Ppy

Zn atoms were inserted into the porphyrin structure by coordinating with the four N atoms in the porphyrin ring to obtain Zn—Ppy. Zinc acetate (41.3 mg, 0.189 mmol) was added to a stirred solution of free porphyrin (50 mg, 0.063 mmol) dissolved in dry DMF (40 mL). Next, the mixed solution was bubbled with Ar for 30 min to relieve the O_2_ present in the reaction system. Then, the solution was heated to reflux with a chilling system for 10 h at 150 °C. When the solution was cooled to room temperature, the solvent was removed in a flash evaporator, and 25 mL of distilled water was added to the reaction mixture. The precipitated powder was centrifuged and washed several times to remove the unreacted zinc acetate and dried overnight in vacuum to obtain Zn—Ppy.

### Synthesis of COF Composites

First the synthesized Zn—Ppy (85.415 mg, 0.1 mmol) and BD (55.83 mg, 0.3 mmol) were dissolved in a mixed solution of dichlorobenzene (20 mL), butanol (20 mL), and acetic anhydride (10 mL) inside a reactor. The mixed solution was purged with Ar for 30 min and heated to 120 °C; the reaction was allowed to continue for 72 h under the reflux system. The cooled product was collected by filtration and thoroughly washed with a mixture of methanol and chloroform (1:1 v/v). Next it was subjected to Soxhlet extraction for 24 h with the same solvent mixture. Finally, the obtained powder products were dried in a vacuum oven for 12 h to yield BD—COF. The BPDA—COF samples were also synthesized by a similar process, except in this case, the ligand was replaced with BPDA.

### Synthesis of the BPDA—COF—TiO_2_ Samples

The synthesized BPDA—COF (100 mg) and titanium isopropoxide (0.25 ml, 0.25 mmol) were refluxed in a mixed solution of dichlorobenzene (20 mL) and butanol (20 mL) at 120 °C for 72 h. The resultant solid after cooling was transferred into a stainless‐steel autoclave, and then heated at 150 °C for 15 h in a furnace for hydrolysis process. Finally, the obtained powder products were collected through centrifugation and washed several times with ethanol and water to produce pure BPDA—COF—TiO_2_ heterostructures. For convenient comparison, BPDA—COF—TiO_2_ composites with different TiO_2_ dosages were prepared by a similar method but varying the amount of TTIP in the reaction. The corresponding samples were named BPDA—COF—TiO_2_‐*x*, where *x* is the percentage of calculated TiO_2_ dosage for the composite preparation.

### Synthesis of the BD—COF—TiO_2_ Samples

The synthesized BD—COF (100 mg) and titanium isopropoxide (0.25 mL, 0.25 mmol) were refluxed in a mixed solution of dichlorobenzene (20 mL) and butanol (20 mL) at 120 °C for 72 h. The resultant solid after cooling was transferred into a stainless‐steel autoclave, and then heated at 150 °C for 15 h in a furnace for hydrolysis process. Finally, the obtained powder products were collected through centrifugation and washed several times with ethanol and water to produce pure BD—COF—TiO_2_ heterostructures.

### Computational Methods

The initial crystal structures of BPDA—COF and BD—COF were built using the Materials Visualizer interface of BIOVIA Materials Studio 2017 (17.2), and these structures along with the cell unit were optimized. The Perdew–Burke–Ernzerh exchange‐correlation functional of generalized gradient approximation (GGA‐PBE) with Grimme's default DFT‐*D* parameters was used. The double‐numeric basis set (DND) was chosen. All calculations were performed using the DMol^3^ module of BIOVIA Materials Studio 2017 (17.2). The optimized crystals were all *P4* space groups with *a* = *b* = 29.915 Å, *α* = *β* = *γ* = 90° for BPDA—COF and *a* = *b* = 30.052 Å, *α* = *β* = *γ* = 90° for BD—COF. The *c*‐axis was long enough to avoid interactions between layers (about 30 Å after optimization). The convergence criteria for structure optimization were set to a) a self‐consistent field (SCF) tolerance of 1 × 10^−5^ Hartree, b) an energy tolerance of 1 × 10^−5^ Hartree, c) a maximum force tolerance of 2 × 10^−3^ Hartree Å^−1^, d) a maximum displacement tolerance of 5 ×10^−3^ Å, and e) Monkhorst–Pack grid *k*‐points of 1 × 1 × 1. Ti_16_O_32_ cluster was optimized with the same method. The optimized Ti_16_O_32_ cluster was put the pore of BPDA—COF and BD—COF to form BPDA—COF—TiO_2_ and BD—COF—TiO_2_ crystals. The geometries of BPDA—COF—TiO_2_ and BD—COF—TiO_2_ crystals were then fully optimized with the same method but keeping the cell parameter unchanged. Based on the optimized crystal structures, the electron properties were performed with a similar setup as optimization, except that the energy tolerance was increased to 1 × 10^−6^ Hartree; DND basis set was changed to the double numeric with polarization functions basis set (DNP); and the *k*‐points were changed to 2 × 2 × 1.

## Conflict of Interest

The authors declare no conflict of interest.

## Supporting information

Supporting Information

## Data Availability

The data that support the findings of this study are available from the corresponding author upon reasonable request.
