# Supplementary material for: Synthesis of Pore‐Wall‐Modified Stable COF/TiO2 Heterostructures via Site‐Specific Nucleation for an Enhanced Photoreduction of Carbon Dioxide
Source: Adv Sci (Weinh). 2023 Mar 25;10(14):2300073. doi: 10.1002/advs.202300073 (PMC10190585; doi:10.1002/advs.202300073)
Supplement: Supplementary file 1 — Supporting Information [file ADVS-10-2300073-s001.pdf]

Supporting Information

Synthesis of Pore-Wall-Modified Stable COF/TiO_2 Heterostructures via Site-Specific Nucleation for an Enhanced Photoreduction of Carbon Dioxide

A. Putta Rangappa,[a] D. Praveen Kumar,[a] Khai H. Do,[a] Jinming Wang,[a] Yuexing Zhang,[b] and Tae Kyu Kim [a]**

[a]A. P. Rangappa, Dr. D. P. Kumar, K. H. Do, Dr. J. Wang, and Prof. T. K. Kim

Department of Chemistry, Yonsei University, Seoul 03722, Republic of Korea

E-mail: tkkim@yonsei.ac.kr

[b]Prof. Y. Zhang

College of Chemistry and Chemical Engineering, Dezhou University, Dezhou 253023, China

E-mail: zhangyuexing@sdu.edu.cn

Characterization details

The photocatalysts were characterized by PXRD, XPS, TEM, DRS, and PL spectrometry. The XRD patterns were collected on a Bruker D8 Advance diffractometer using Cu Kα radiation. The XPS measurements were performed using Al Kα radiation (1486.6 eV) with an energy of 15 kV/150 W. The TEM and high-resolution TEM images were obtained using a Jeol JEM-ARM200F system operated at an acceleration voltage of 200 kV. The DRS spectra were recorded on a Shimadzu UV-1800 system, and the PL properties were studied using a Hitachi F-7000 fluorescence spectrometer. The TRFS were measured using a femtosecond fluorescence spectrophotometer (Edinburgh FES 920). In-situ diffuse reflectance infrared Fourier transform spectroscopy (DRIFTS) were recorded at a Nicolet iS50 spectrometer with HgCdTe (MCT/A) liquid nitrogen cooling detector under 4 cm^{-1} resolutions by using 64 scans. PHI5000 Versaprobe III XPS equipped with a monochromatic Al Kα source with an energy of 1486.6 eV was used for the in-situ XPS analysis. The samples were analyzed in CAE scan mode while a vacuum of $\leq 4.78\times10^{-6}$ Pa was maintained.

The photoelectrochemical measurements were carried out using a CHI 617B electrochemical analyzer and three-electrode system, with Pt wire and Ag/AgCl as the counter electrode and reference electrodes, respectively. The working electrode was prepared by spinning a photocatalyst suspension containing Nafion solution (30 μL, 0.02 mg μL^{-1}) onto an In-doped SnO_2-coated glass, which was then placed in an oven at 100 °C for 3 h to dry the solvent and solidify the samples. The photocurrent curves were measured in Na_2SO_4 (electrolyte; 0.5 mol L^{-1}) during the on/off period of the 150-W Xe lamp solar simulator equipped with an AM 1.5G filter. Mott-Schottky experiments were carried out in the dark at frequencies of 200 and 500 Hz using the same procedure as described above.

Photocatalytic performance

The CO_2 photoreduction experiments were carried out in a 210 mL enclosed glass reactor with a quartz window. In a typical test, 2 mg of solid catalyst was dispersed in 10 mL of acetonitrile/BIH solution (0.5 g L^{-1}). The reactor was vacuumed and purged with high purity CO_2 for 5 min, respectively, which was repeated for two times. A 150-W Xe lamp (Abet Technologies) equipped with an AM 1.5G filter was used as the light source for the photocatalytic experiments. The output light intensity was adjusted to 1 sun (100 mW cm^{-2}) using a standard Si reference cell (Abet Technologies). The photoreduction products were analyzed by off-line gas chromatography using a gas chromatograph (Agilent 8890, TDX-01 column) equipped with two independent detectors to detect H_2 (thermal conductivity detector) and CH_4/CO (flame ionization detector), and the product amounts were calibrated by an external standardization method with a commercial standard gas mixture.

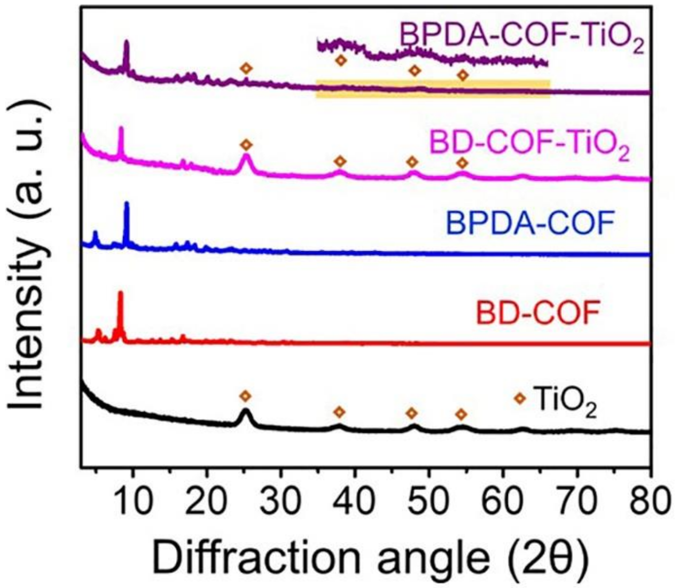

Figure S1. (a) XRD patterns of TiO_2, COF, and the COF/TiO_2 heterostructures.

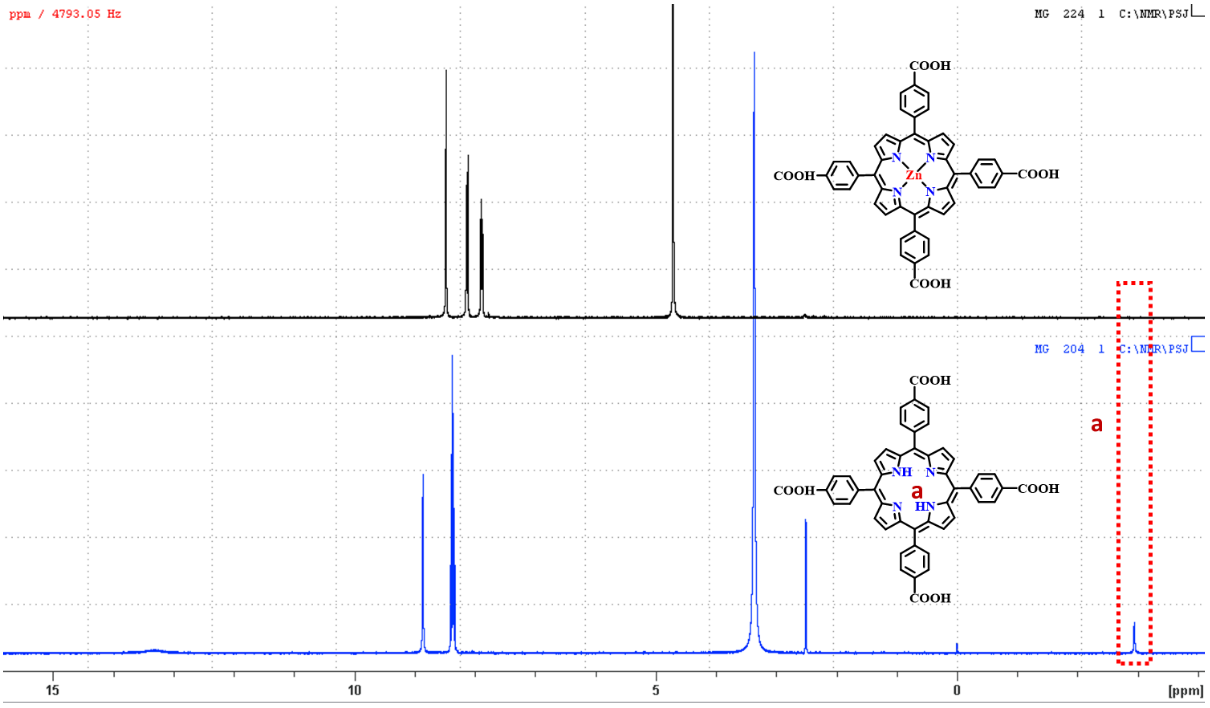

Figure S2. NMR spectra of the free Ppy and Zn-Ppy.

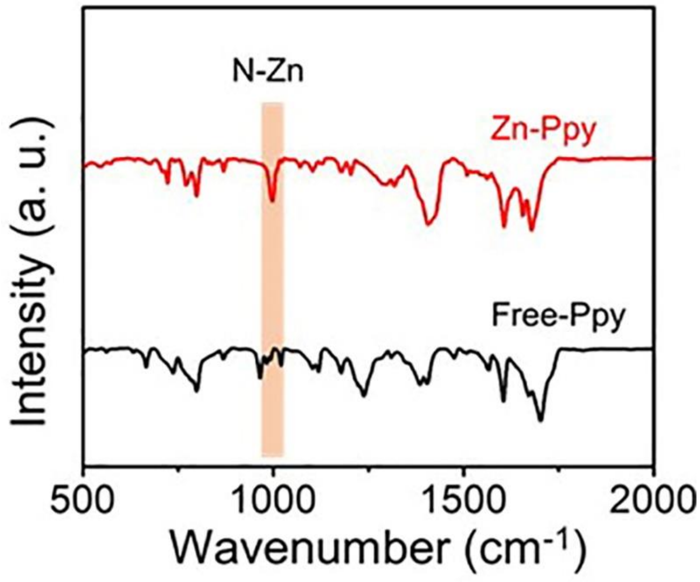

Figure S3. FTIR spectra of the free Ppy and Zn-Ppy.

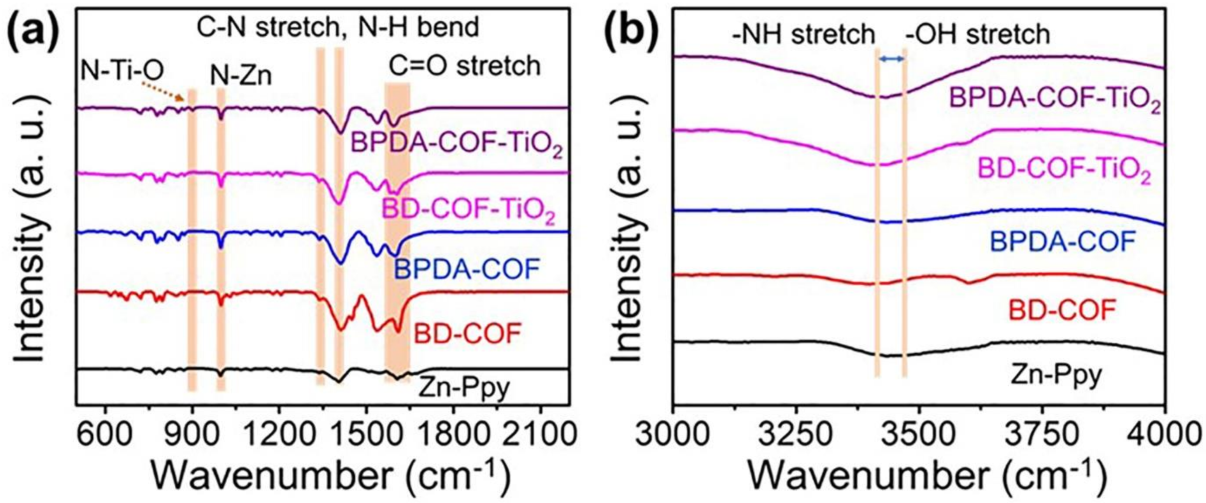

Figure S4. FTIR spectra of Zn-Ppy, COF, and the COF/TiO_2 heterostructures.

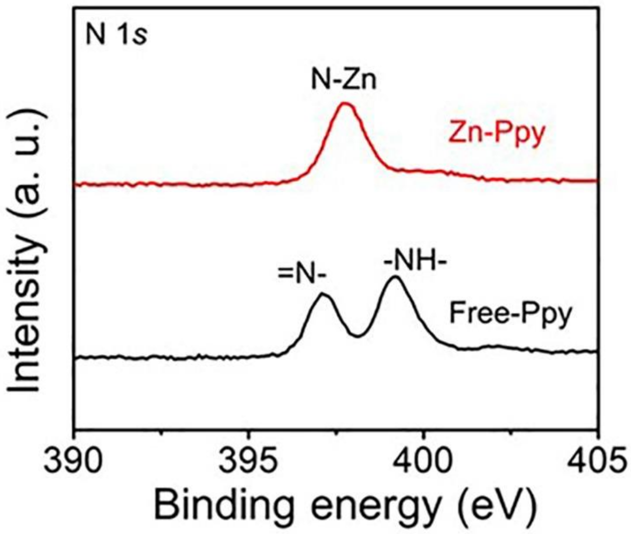

Figure S5. High-resolution N 1s XPS profiles of free Ppy and Zn-Ppy.

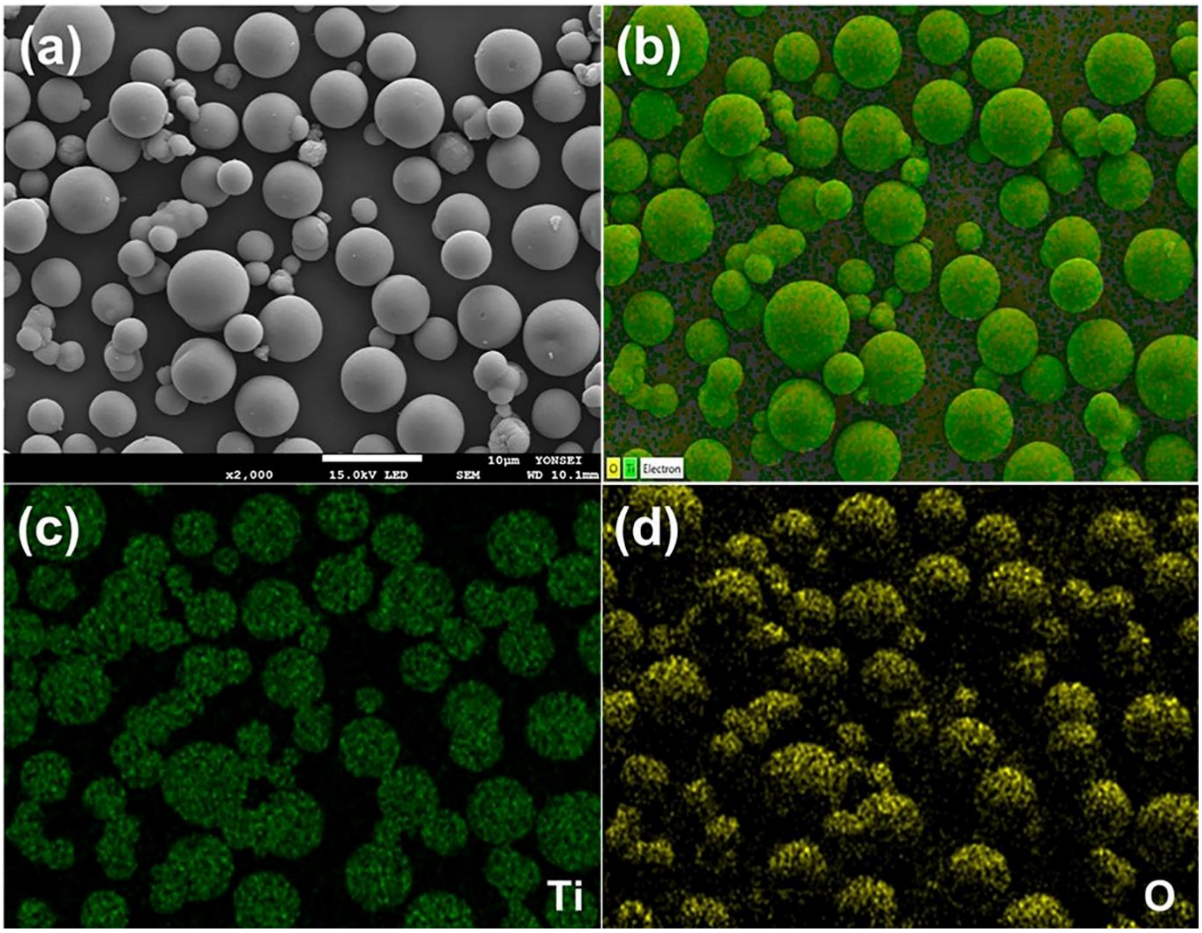

Figure S6. (a) FESEM image of the synthesized TiO_2 samples, and (b–d) their corresponding EDX elemental mappings.

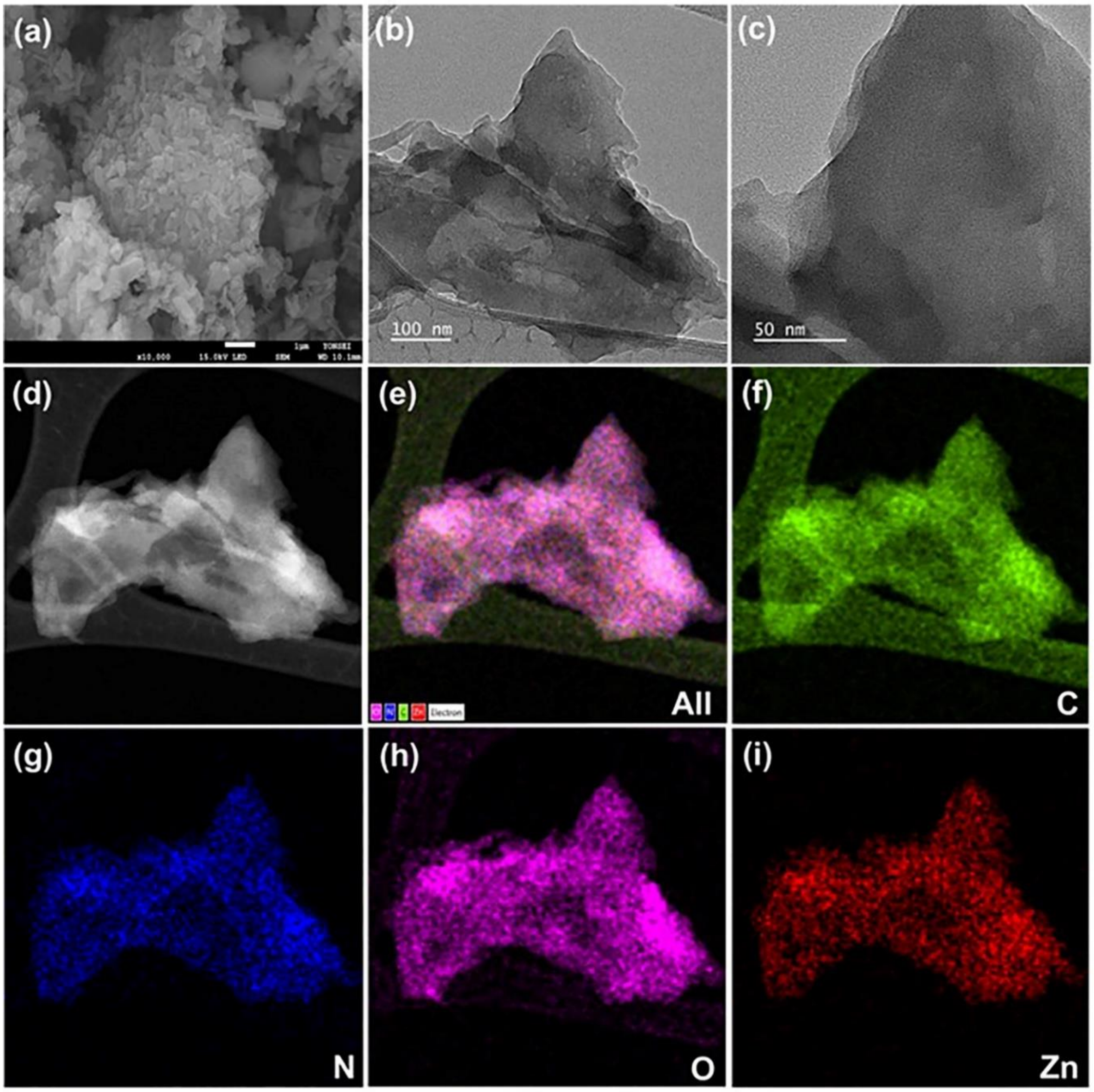

Figure S7. (a) FESEM image, (b–d) TEM images, and (e–i) the corresponding EDX elemental mappings of the BPDA-COF nanocomposite.

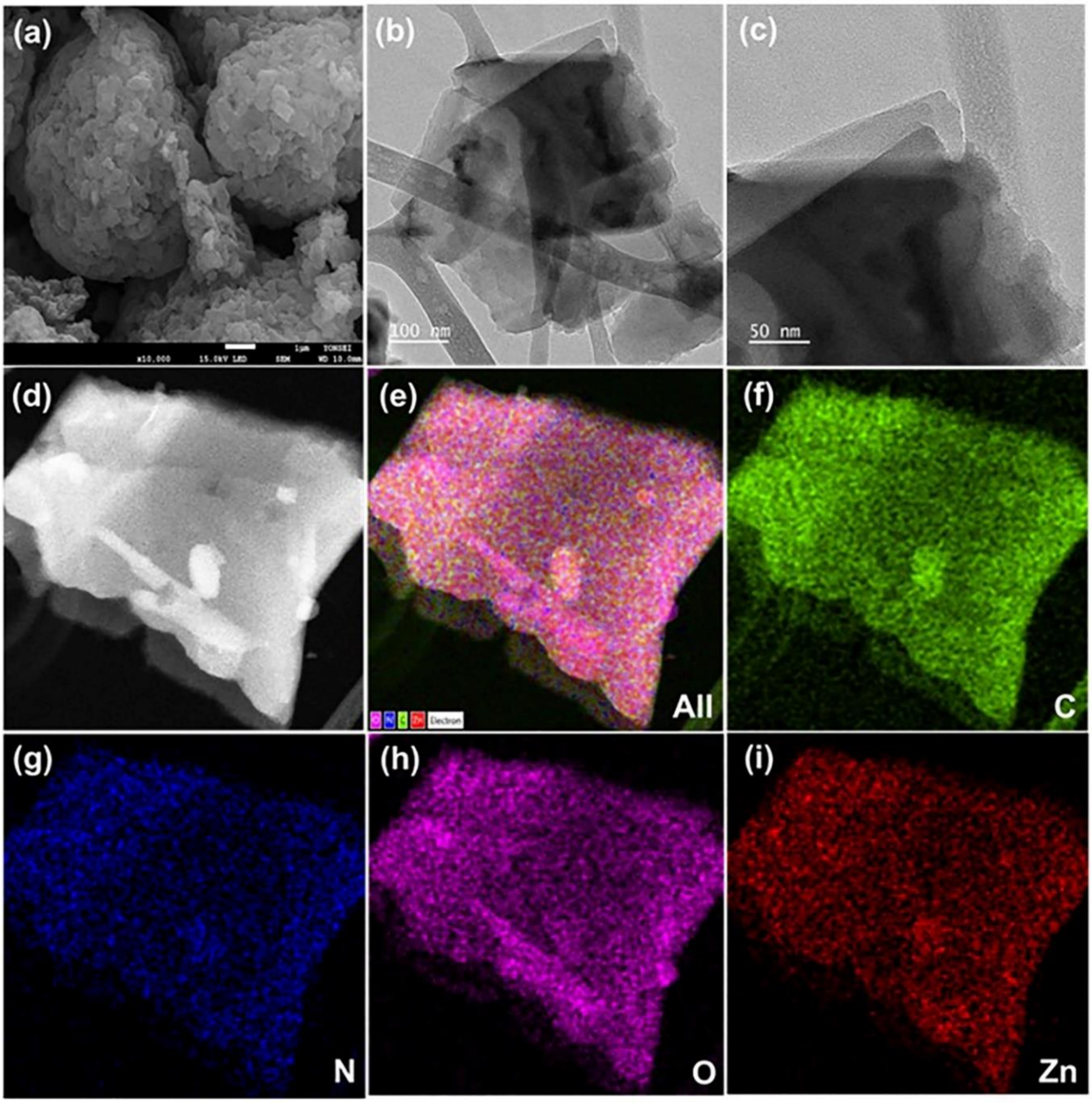

Figure S8. (a) FESEM image, (b–d) TEM images, and (e–i) the corresponding EDX elemental mappings of the BD-COF nanocomposite.

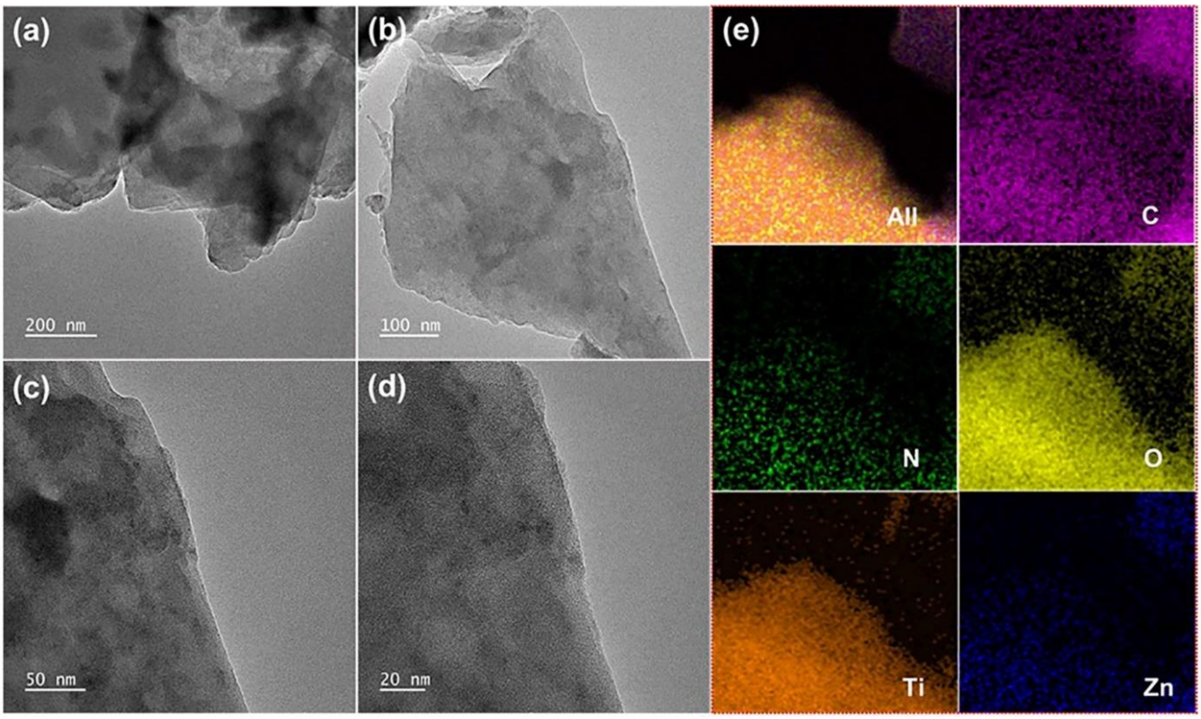

Figure S9. (a–d) TEM images and (e) corresponding EDX elemental mappings (C, N, Ti, O, and Zn) of the BPDA-COF-TiO_2 composite.

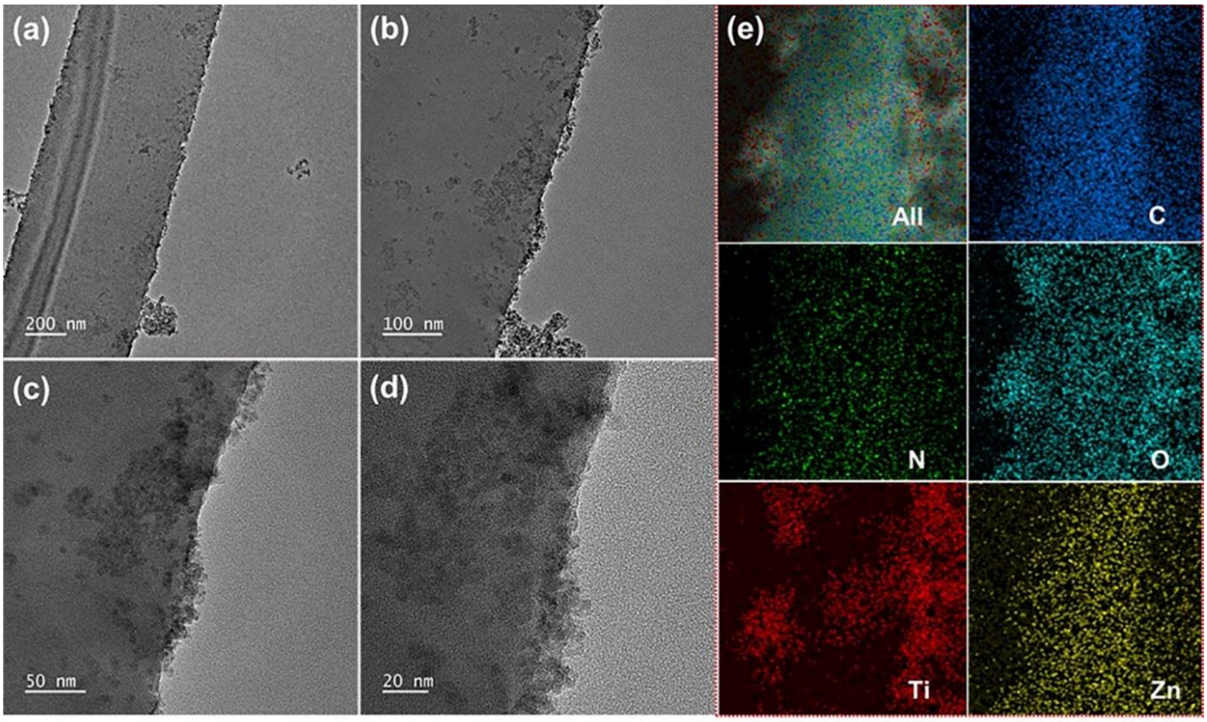

Figure S10. (a–d) TEM images and (e) corresponding EDX elemental mappings (C, N, Ti, O, and Zn) of the BD-COF-TiO_2.

Figure S11. Control experiments to optimize the reaction medium for selective CO_2 reduction to CO.

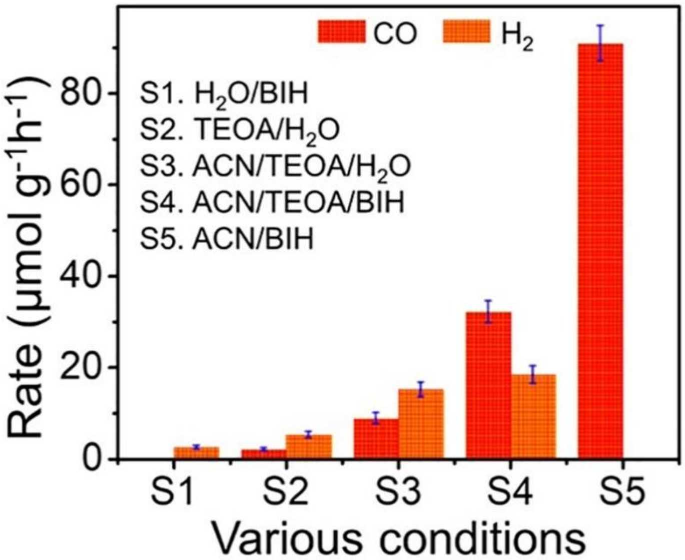

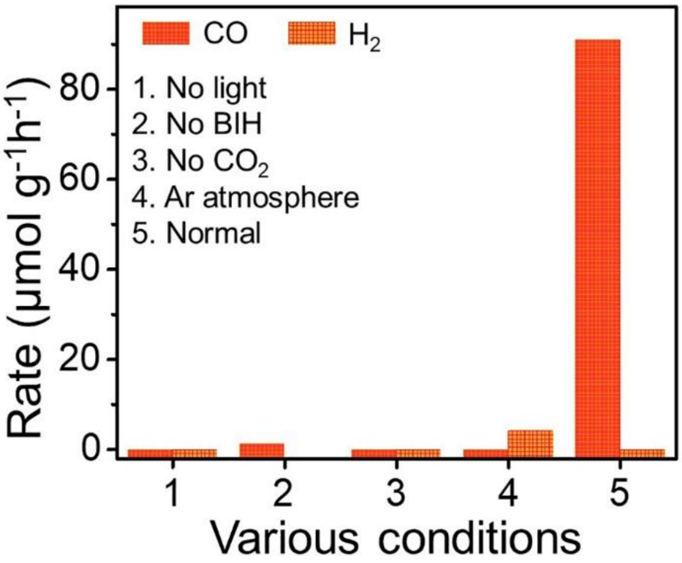

Figure S12. Comparative experiments proving the obtained CO originated from the photoreduction of CO_2.

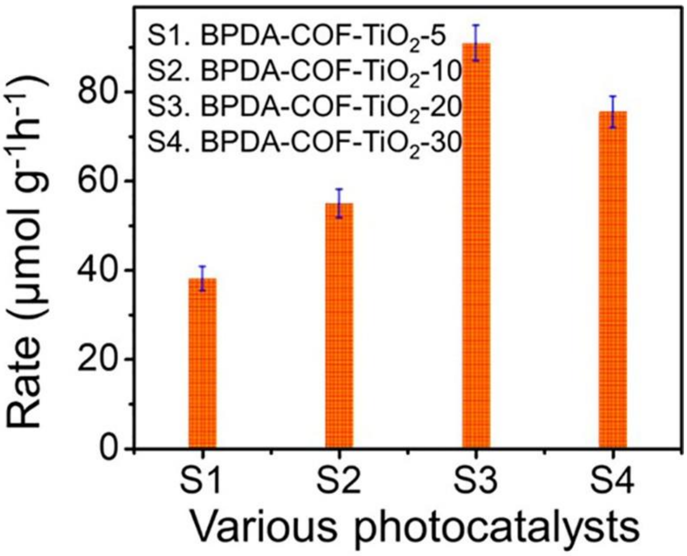

Figure S13. Average rate of CO generation by BPDA-COF with different TiO_2 (%) dosage irradiated for 5 h.

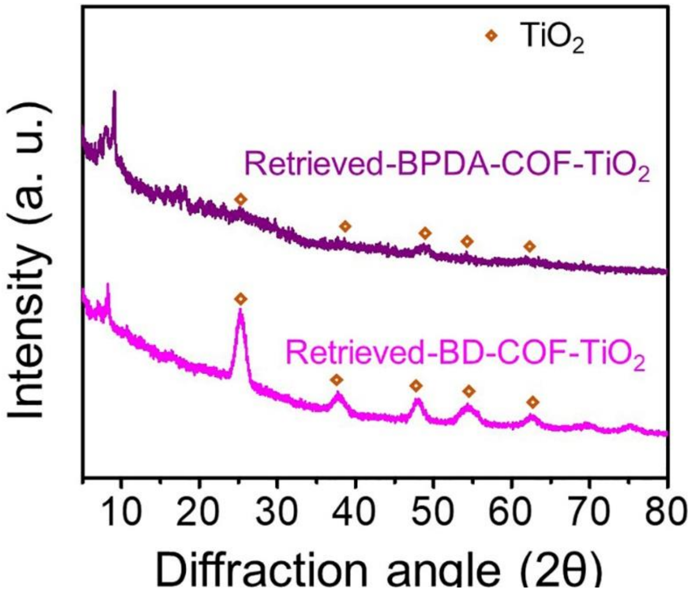

Figure S14. XRD spectra of BD-COF-TiO_2 and BPDA-COF-TiO_2 after long time photocatalytic measurements.

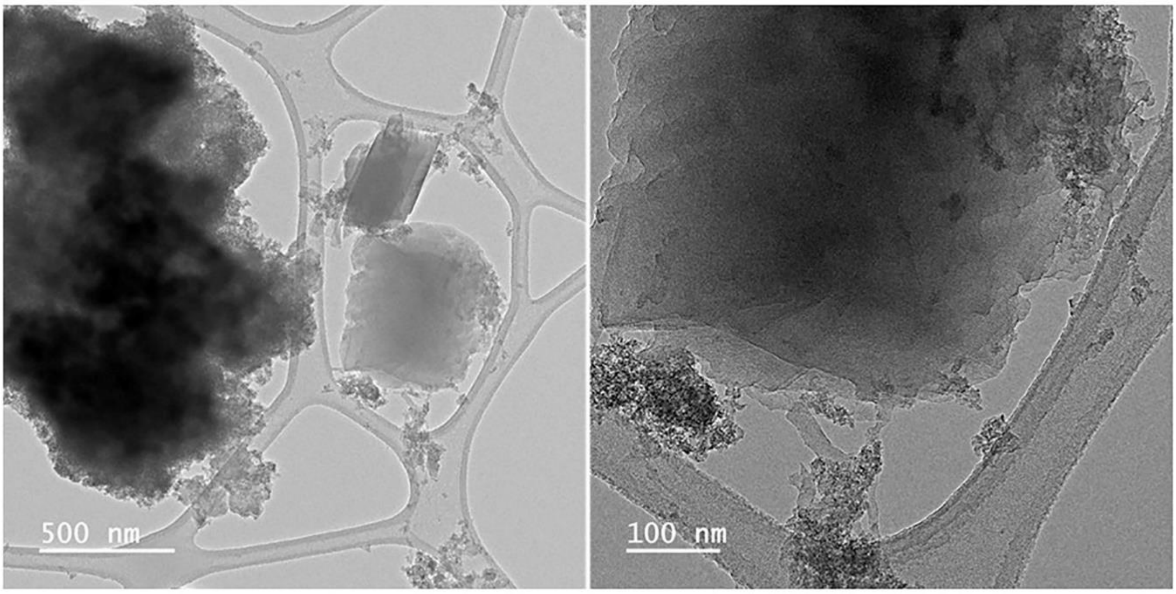

Figure S15. TEM images of the BD-COF-TiO_2 composite after long time photocatalytic measurements.

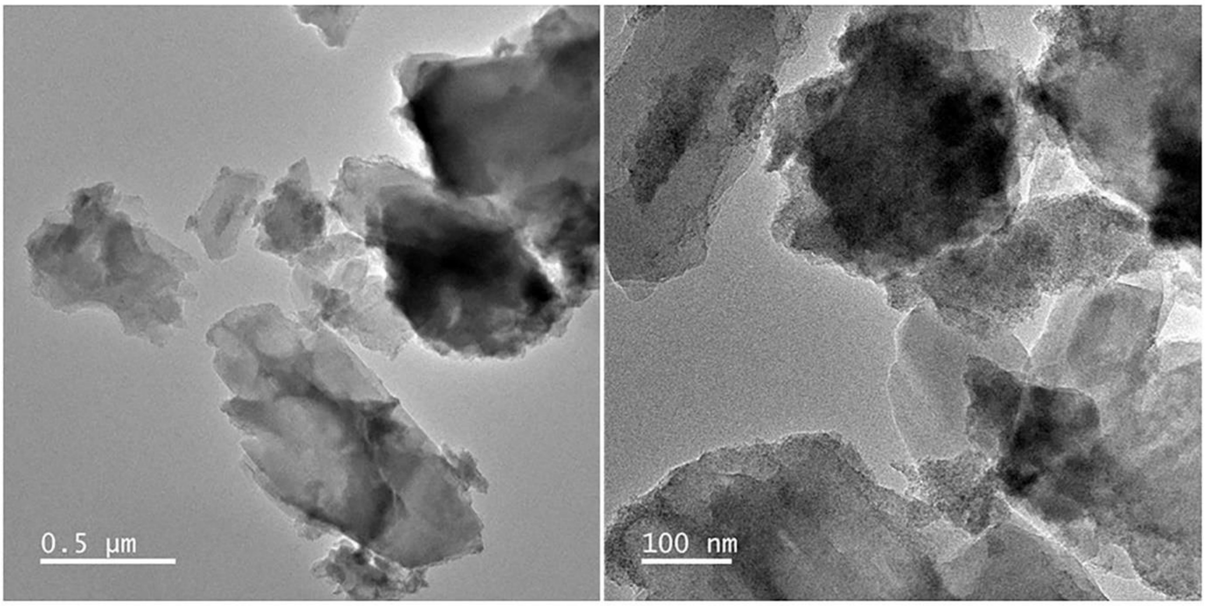

Figure S16. TEM images of the BPDA-COF-TiO_2 composite after long time photocatalytic measurements.

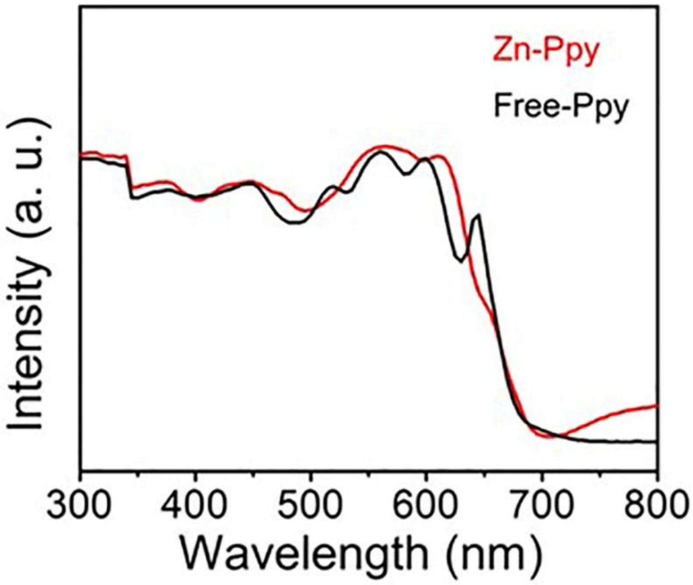

Figure S17. DRS absorption spectra of free Ppy and Zn-Ppy.

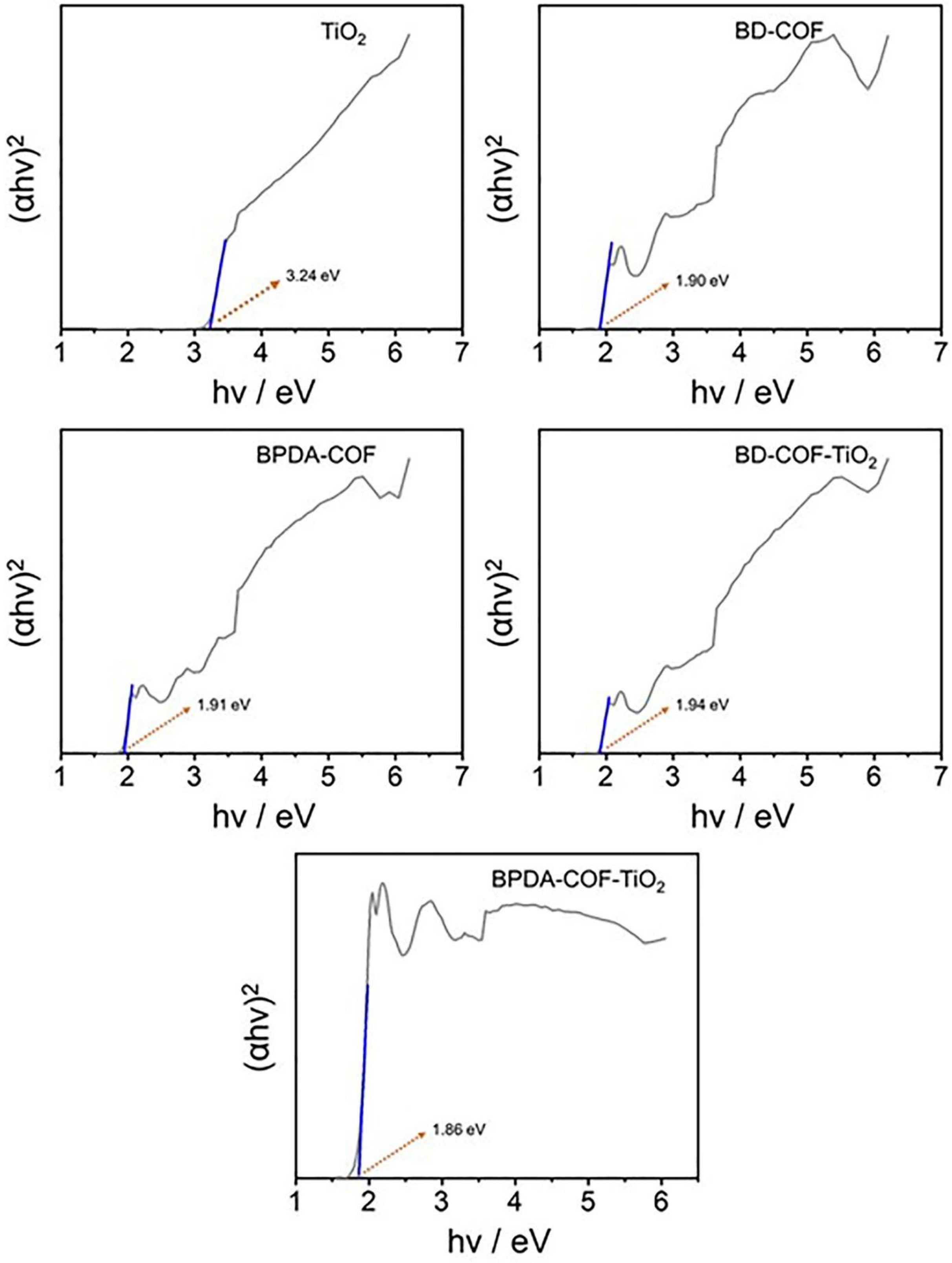

Figure S18. Tauc plots derived from the corresponding DRS absorption spectra.

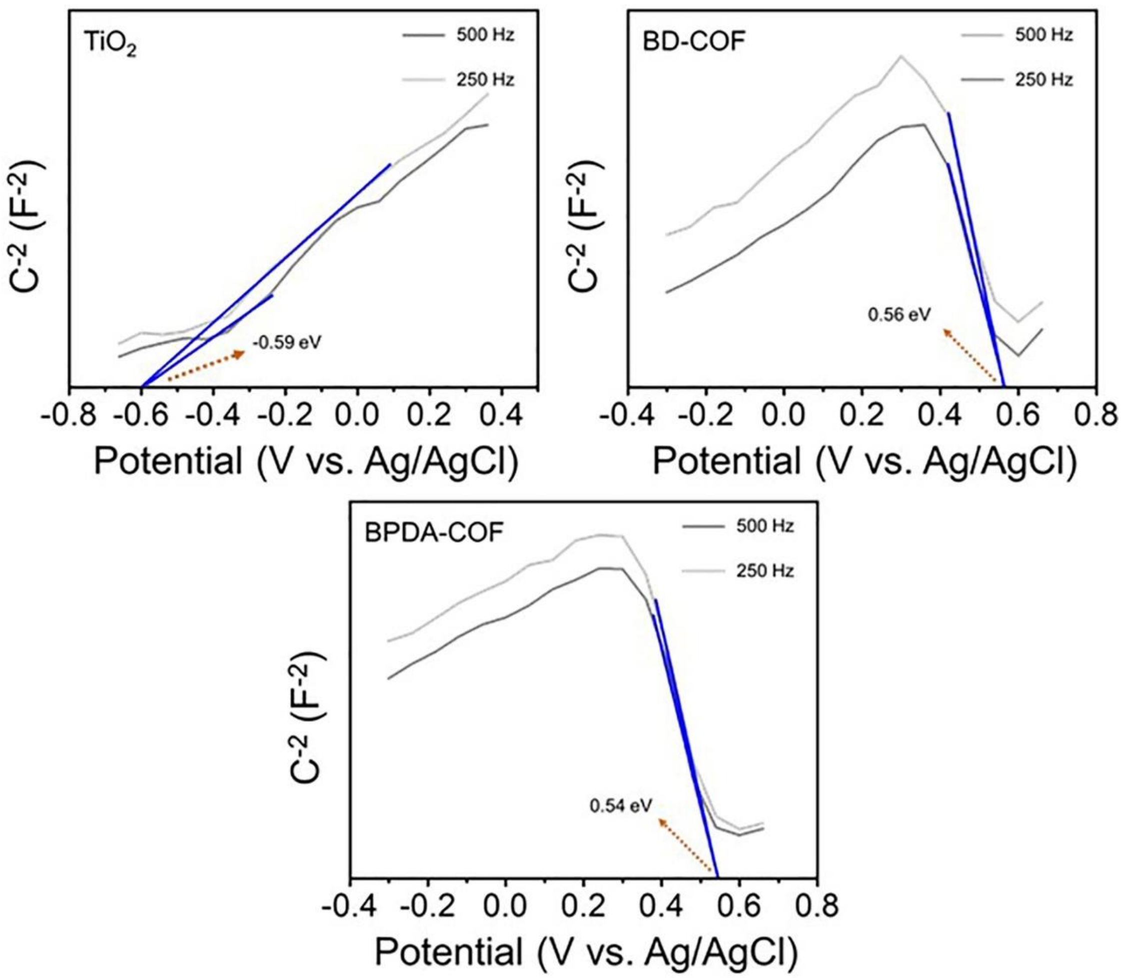

Figure S19. Mott–Schottky analysis of the TiO_2 and COF nanocomposites.

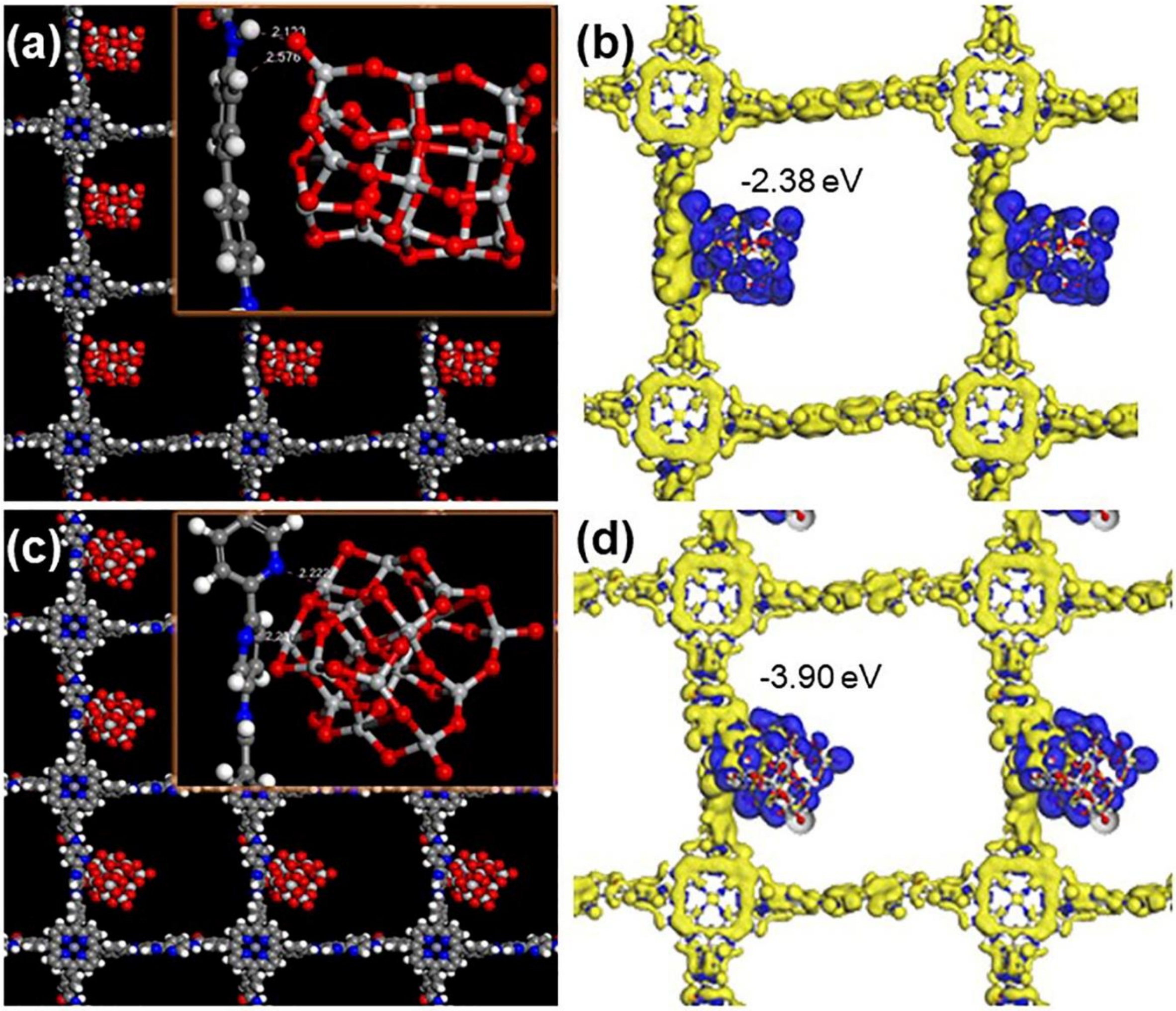

Figure S20. Optimized geometry (enlarged region in inset) and 3D charge distribution of the (a, b) BD-COF-TiO_2 and (c, d) BPDA-COF-TiO_2 heterostructures (number in b, d indicates the BE between the COF and TiO_2).

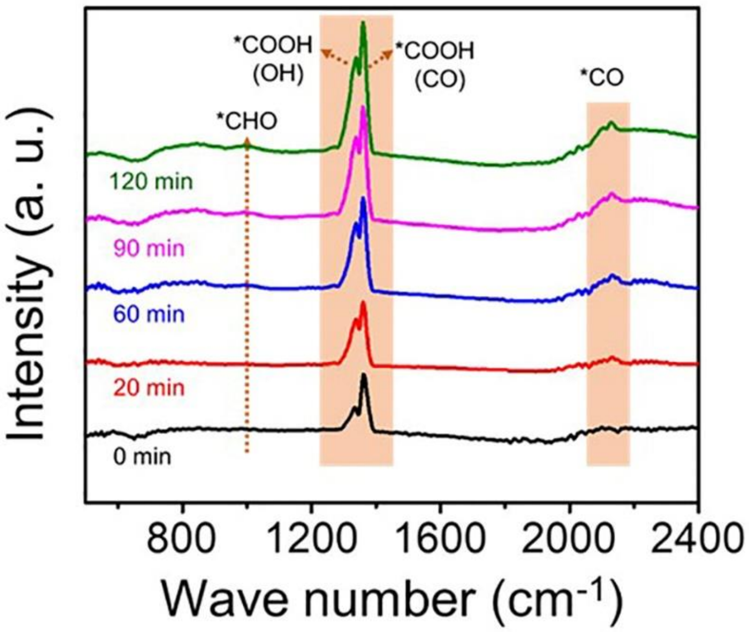

Figure S21. DRIFT analysis for the BPDA-COF-TiO_2 composite.

Table S1. Comparisons of this work and previously reported photocatalytic CO_2 reduction rates of COF and TiO_2 related photocatalysts.

Photocatalyst	Reaction conditions	Light source	Production rate (μmol g^{-1} h^{-1})	Ref.
BD-COF	2mg (ACN/BIH)	150 W Xe lamp (Full spectrum: 1 sun)	CO: 14	This work
BD-COF-TiO_2			CO: 46	
BPDA-COF			CO: 22	
BPDA-COF-TiO_2			CO: 91	
CoPor-RuN_3	5 mg (ACN/BIH)	300 W Xe lamp ($\lambda \geq 400$ nm)	CO: 37.1 CH_4: 1.57	S1
TTCOF-Zn	100 mg (Water)	300 W Xe lamp ($\lambda \geq 400$ nm)	CO: 2.0	S2
TiO_2-INA@CuP-Ph COF	100 mg (Water)	300 W Xe lamp ($\lambda \geq 400$ nm)	CO: 50.5	S3
PD-HPP-TiO_2	30 mg (Water)	300 W Xe lamp (Visible light)	CO: 34 CH_4: 48	S4
COF-TiO_2	20 mg (Water)	300 W Xe lamp (800 nm $\geq \lambda \geq$ 380 nm)	CO: 69.67	S5
COF-Bi_2WO_6			CO: 57.1	
COF-αFe_2O_3			CO: 31.43	
Ru-TpPa-1	15 mg (ACN/TEOA)	300 W Xe lamp (800 nm $\geq \lambda \geq$ 420 nm)	HCOOH: 108.8	S6
TiO_2/COF	20 mg (ACN/Water/TEOA/2,2′-bipyridine/$CoCl_2$)	300 W Xe lamp	CO: 580 H_2: 29	S7
Ni-TpBPy	10 mg (ACN/Water/TEOA/ $[Ru(bpy)_3]Cl_2\cdot 6H_2O$)	300 W Xe lamp ($\lambda \geq 400$ nm)	CO: 811.4 H_2: 32.4	S8
Sp^2C-COF_{dpy}-Co	20 mg (Water/TEOA)	300 W Xe lamp ($\lambda \geq 400$ nm)	CO: 996 H_2: 185.2	S9
PdIn@N_3-COF	2 mg (Water)	300 W Xe lamp (400 nm cut off)	CH_3OH: 24.6 Ethanol: 8.7	S10
Ni-PCD@TD-COF	5 mg (ACN/Water/TEOA/ $[Ru(bpy)_3]Cl_2\cdot 6H_2O$)	Xe lamp ($\lambda \geq 400$ nm)	CO: 478 H_2: 9.5	S11

ACN: Acetonitrile, BIH: 1,3- dimethyl-2-phenyl-2,3-dihydro-1H-benzo[d]imidazole, TEA: Triethylamine, TEOA: Triethanolamine, and COF: Covalent organic framework

References

[S1] S. Chen, P. King, H. Niu, H. Liu, X. Wang, J. Zhang, R. Li, Y. Guo, T. Peng, *Chem. Eng. J.* **2022,** 431, 133357.

[S2] M. Lu, J. Liu, Q. Li, M. Zhang, M. Liu, J. L. Wang, D. Q. Yuan, Y. Q. Lan, *Angew. Chem. Int. Ed.* **2019,** 131, 12522–12527.

[S3] L. Wang, G. Huang, L. Zhang, R. Lian, J. Huang, H. She, C. Liu, Q. Wang, *J. Ener. Chem.* **2022,** 64, 85–92.

[S4] Y. Ma, X. Yi, S. Wang, T. Li, B. Tan, C. Chen, T. Majima, E. R. Waclawik, H. Zhu, J. Wang, *Nat. Commun.* **2022,** 13, 1400.

[S5] M. Zhang, M. Lu, Z. L. Lang, J. Liu, M. Liu, J. N. Chang, L. Y. Li, L. J. Shang, M. Wang, S. L. Li, Y. Q. Lan, *Angew. Chem. Int. Ed.* **2020,** 59, 6500–6506.

[S6] K. Guo, X. Zhu, L. Peng, Y. Fu, R. Ma, X. Lu, F. Zhang, W. Zhu, M. Fan, *Chem. Eng. J.* **2021,** 405, 127011.

[S7] X. An, J. Bian, K. Zhu, R. Liu, H. Liu, J. Qu, *Chem. Eng. J.* **2022,** 442, 135279.

[S8] W. Zhong, R. Sa, L. Li, Y. He, L. Li, J. Bi, Z. Zhuang, Y. Yu, Z. Zou, *J. Am. Chem. Soc.* **2019,** 141, 7615–7621.

[S9] Y. Xiang, W. Dong, P. Wang, S. Wang, X. Ding, F. Ichihara, Z. Wang, Y. Wada, S. Jin, Y. Weng, H. Chen, H. Chen, J. Ye, *Appl. Catal. B* **2020,** 274, 119096.

[S10] Y. Huang, P. Du, W. X. Shi, Y. Wang, S. Yao, Z. M. Zhang, T. B. Lu, X. Lu, *Appl. Catal. B* **2021,** 288, 120001.

[S11] H. Zhong, R. Sa, H. Lv, S. Yang, D. Yuan, X. Wang, R. Wang, *Adv. Funct. Mater.* **2020,** 30, 2002654.
